# Recently Approved Drugs for Lowering and Controlling Intraocular Pressure to Reduce Vision Loss in Ocular Hypertensive and Glaucoma Patients

**DOI:** 10.3390/ph16060791

**Published:** 2023-05-26

**Authors:** Najam A. Sharif

**Affiliations:** 1 Eye-APC Duke-NUS Medical School, Singapore 169856, Singapore; gmsnas@nus.edu.sg or najsharif777@gmail.com; 2 Singapore Eye Research Institute, Singapore 169856, Singapore; 3 Department of Pharmacology and Neuroscience, University of North Texas Health Sciences Center, Fort Worth, TX 76107, USA; 4 Department of Pharmacy Sciences, Creighton University, Omaha, NE 68178, USA; 5 Department of Pharmaceutical Sciences, College of Pharmacy and Health Sciences, Texas Southern University, Houston, TX 77004, USA; 6 Imperial College of Science and Technology, St. Mary’s Campus, London SW7 2BX, UK; 7 Institute of Ophthalmology, University College London, London WC1E 6BT, UK

**Keywords:** eye pressure, eyesight, glaucoma, intraocular pressure, ocular hypertension, therapeutic modalities, IOP-lowering, drugs, microshunt

## Abstract

Serious vision loss occurs in patients affected by chronically raised intraocular pressure (IOP), a characteristic of many forms of glaucoma where damage to the optic nerve components causes progressive degeneration of retinal and brain neurons involved in visual perception. While many risk factors abound and have been validated for this glaucomatous optic neuropathy (GON), the major one is ocular hypertension (OHT), which results from the accumulation of excess aqueous humor (AQH) fluid in the anterior chamber of the eye. Millions around the world suffer from this asymptomatic and progressive degenerative eye disease. Since clinical evidence has revealed a strong correlation between the reduction in elevated IOP/OHT and GON progression, many drugs, devices, and surgical techniques have been developed to lower and control IOP. The constant quest for new pharmaceuticals and other modalities with superior therapeutic indices has recently yielded health authority-approved novel drugs with unique pharmacological signatures and mechanism(s) of action and AQH drainage microdevices for effectively and durably treating OHT. A unique nitric oxide-donating conjugate of latanoprost, an FP-receptor prostaglandin (PG; latanoprostene bunod), new rho kinase inhibitors (ripasudil; netarsudil), a novel non-PG EP2-receptor-selective agonist (omidenepag isopropyl), and a form of FP-receptor PG in a slow-release intracameral implant (Durysta) represent the additions to the pharmaceutical toolchest to mitigate the ravages of OHT. Despite these advances, early diagnosis of OHT and glaucoma still lags behind and would benefit from further concerted effort and attention.

## 1. Introduction

The aim of this review is to present the pharmacological characteristics, in vivo efficacy, and side-effect profiles of drugs that were approved by global health authorities within the last six years to treat ocular hypertension and open-angle glaucoma. Pre-clinical and clinical data will be appraised, and a brief discourse on future prospects will also be presented.

Blindness is undoubtedly the worst human sense-related ailment that a person has to deal with and endure during their lifetime. Even less severe and dire outcomes due to visual impairment can inflict psychological pain due to feelings of helplessness, loneliness, and alienation that cause social disengagement and isolation with a profound loss in quality of life [1,2,3,4]. Indeed, much has been written about the debilitating eye disease called “glaucoma” and how this silent thief of sight robs millions of people worldwide of their eyesight, even though it is largely preventable [5,6,7,8]. Glaucoma is a constellation of degenerative eye conditions in which the major risk factors include advancing age, elevated intraocular pressure (IOP), African–American ancestry, low intracranial fluid pressure, and reduced retinal blood flow [5,6,7,8]. These features, coupled with other insidious endogenous toxic factors, damage the optic nerve and retinal ganglion cells (RGCs) to cause vision loss that can, over several years, render the afflicted patient blind unless a diagnosis of elevated IOP (ocular hypertension, OHT) and glaucomatous optic neuropathy (GON) occurs via an in-depth eye examination and suitable treatment is initiated. 

Essentially, there are five forms of glaucoma classified according to certain criteria [1,2,3,4,8]. These are: open-angle (chronic) glaucoma (OAG), angle-closure (acute/chronic) glaucoma (ACG), congenital glaucoma, secondary glaucoma, and normal tension glaucoma (NTG) [9,10,11,12,13,14,15,16,17,18,19,20]. For brevity and focus, only OAG will be discussed since much of the research and approved drug, device, and surgical treatments have been directed towards this disease, although the latter modalities are also utilized for other types of glaucoma. Since several clinical trials have demonstrated a strong association between OAG and elevated IOP/ chronic OHT (cOHT) [9,10,11,12,13,14,15,16,17,18,19,20,21,22], the focus of much research has been to find means to lower and control this modifiable biomarker, thereby slowing the progression of the disease [5,6,7,23,24,25]. 

## 2. Open-Angle Glaucoma

**Figure 1:**
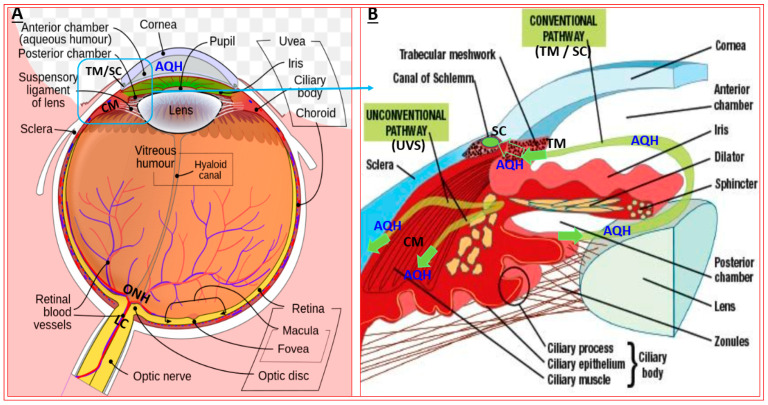
High-level anatomical features of the human eye. The major relevant tissue structures of the eye are shown in A. The inset area outlined in (**A**) is shown in more detail in (**B**) to highlight key elements of the anterior chamber and the routes of AQH flow. Moreover, the tissues and other components involved in the synthesis and drainage of the AQH from the anterior chamber are depicted in (**B**).

**Figure 2:**
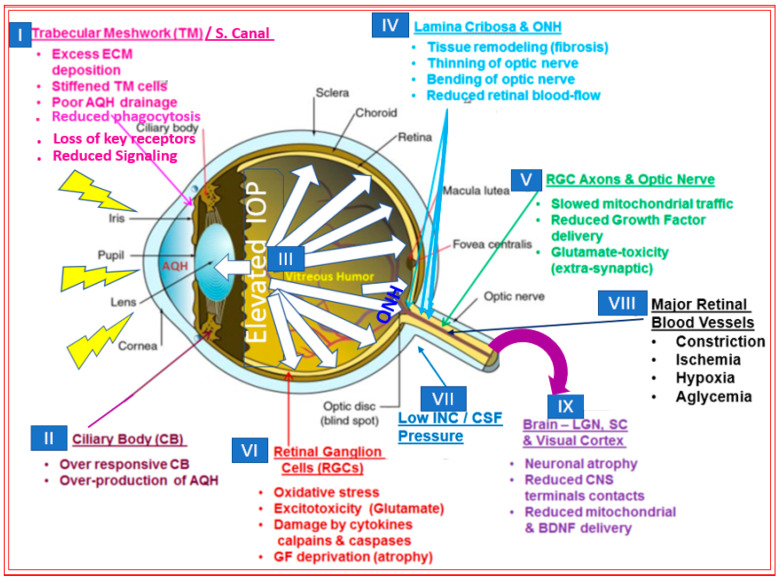
The figure shows the many areas and tissues in the front and back of the eye, the optic nerve and brain relay stations, and visual cortex impacted by diverse sets of events and chemical factors during development of glaucomatous optic neuropathy that can be induced by elevated IOP (ocular hypertension). AQH, aqueous humor; CSF, cerebrospinal fluid; INC, intracranial; LGN, lateral geniculate nucleus; ONH, optic nerve head; SC, Schlemm’s canal.

**Figure 3:**
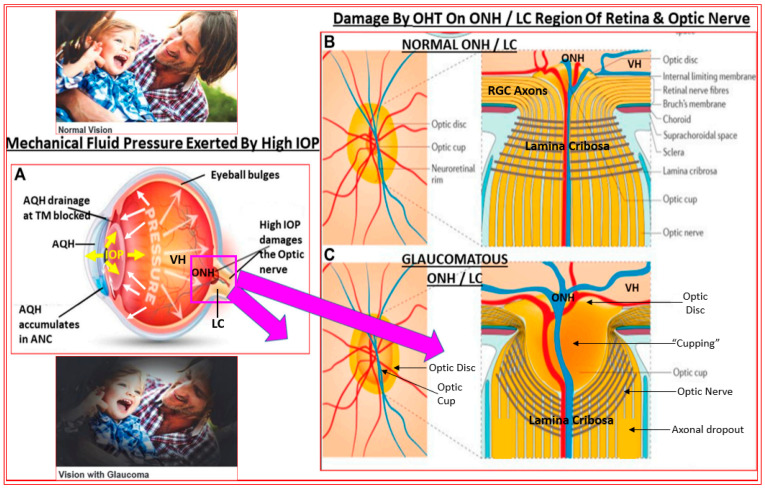
This figure highlights how the raised IOP in the anterior chamber of the eye exerts mechanical compressive pressure on the optic nerve head (ONH) and lamina cribosa (LC) regions at the retinal and optic nerve levels (**A**,**B**). The many aspects of damage to the LC result in retinal ganglion cell axon loss, causing reduction in the optic disc tissue and an increase in the cupping (**C**). This culminates in a reduction in RGC axonal connections to the brain and, hence, visual impairment. All these detrimental changes occur imperceptibly, and while the degenerative process occurs over decades, untreated ocular hypertension continues to rob the patient of their peripheral vision (see insets of the two people under normal and glaucomatous conditions). At this point, perhaps over half of the original RGCs and their axons have been destroyed, and the patient is on the dangerous path to becoming blind in the affected eye.

OAG is the most prevalent of the glaucomas worldwide, having already afflicted >53 million people thus far, with further increases predicted to reach >80 million by 2040 [4,5,6,7]. OAG, sometimes referred to as primary open angle glaucoma (POAG), accounts for >70% of all glaucoma cases. The pathology of OAG impacts numerous parts of the eye, optic nerve, brain relay centers, and visual cortex, but often begins with structural and functional dysfunctions within various tissue components of the anterior chamber (AC) of the eye (Figure 1). Summarily, the aqueous humor fluid (AQH) produced by the ciliary epithelial cells of the ciliary body [26] flows through the pupil into the AC, from where it flows towards the trabecular meshwork (TM) [27,28,29,30,31], and Schlemm’s canal (SC) [32,33,34,35] to exit the AC and empty into the blood circulation via distal micro-vessels [27,28,29,30,31,32,33,34,35]. This conventional TM/SC drainage of AQH represents 70–90% of the total egress from the AC [27,28,29,30,31]. Even though unconventional outflow of the AQH via spaces between ciliary muscle bundles and scleral tissue (uveoscleral (UVS) pathway) can occur, this route ordinarily only accounts for about 10–30% of the total AQH drained from the AC [27,36]. The TM/SC pathway provides the major route for AQH egress and creates resistance that regulates IOP and endeavors to prevent IOP spikes [37,38] while maintaining the shape of the eyeball. Due to the heterogeneity of the TM cells (smooth muscle-like, endothelial-like, and fibroblast-like) [30,31], this tissue has numerous functions, including the production and secretion of local neurotrophic factors and proteolytic enzymes, and contributes to many constituents of the extracellular matrix (ECM) such as collagen, fibronectin, proteoglycans, and of course several types of cytokines [26,27,28,29]. With increasing age and pathologically altered homeostasis, the capacity and capability, number, and phagocytic activity of the TM cells decline [39,40,41] and proteins and lipids, cellular organelles, and other debris, which is normally digested and cleared, begin to accumulate in and around the TM, the juxtacanicular area by the SC, and within the ciliary muscle [42,43,44]. The TM cells also become less flexible and have difficulty changing shape to potentially create vacuoles to transport water across their cell membranes and/or generate spaces between the cells to permit AQH to flow through to the SC [45,46,47,48]. There is also the issue of the uncoupling of the transient receptor potential vanilloid cation channels (e.g., TRPV-4) [49,50,51,52] from the nitric oxide (NO)-generating system in TM cells, which express endothelial and/or inducible NO synthase(s) that results in less cGMP being produced [53,54,55] and thus a reduced ability of TM cells to relax in the glaucomatous disease environment. Moreover, under eye disease conditions as in OAG, the ECM elements are abnormally overproduced due to the release of transforming growth factor-β1/2 (TGF-β1/2) which up-regulates connective tissue growth factor (CTGF) [43,44,46,48], with resultant excess deposition of ECM components and mutant myocilin [42]. These congest the TM and SC and restrict AQH outflow, and by killing some of the TM cells [56,57,58,59,60,61,62,63,64,65,66,67,68,69], they increase the overall resistance for AQH to flow out of the anterior chamber. The chronic accumulation of AQH in the AC in the front of the eye steadily raises the IOP, which physically distends all parts of the eye and induces inflammatory reactions along the retina-optic nerve-brain axis [61,62,63,64,65,66], to which the patient remains oblivious. While low levels of inflammation endeavor to provide protection and homeostasis, chronically released pro-inflammatory substances damage many parts of the eye, particularly the optic nerve [61,62,63,64,65,66,67,68]. As can be surmised from the pictograms in Figure 2 and Figure 3, chronic ocular hypertension (cOHT) imperceptibly bulges out the eyeball and transmits compressive pressure at the retinal optic nerve head (ONH) [5,6,7,69,70]. This induces inflammation by transforming local astrocytes into activated microglia at the ONH and at the delicate lamina cribosa (LC) tissue [71,72,73,74,75,76], a meshwork of collagen fibers within the ONH. The LC provides structural and nutritional support to RGC axons, which pass through the pores within this meshwork enroute to the brain. Essentially, the LC is the initial unmyelinated portion of the optic nerve, and the resulting degradation of it by proteases released by the inflammatory events weakens the latter. Consequently, the RGC axons bend and slow down the axonal transport of mitochondria and trophic factors from the brain to the RGC somas and vice versa [77,78,79,80,81,82,83,84,85,86,87,88,89] thereby reducing the communication between the eye and the brain. Soon the retinal blood vessels also exhibit increased tortuosity, and periods of ischemia occur that cause hypoxia and oxidative stress, resulting in mitochondrial dysfunction in the vulnerable RGCs, LC cells, and RGC axons [67,90,91,92,93,94,95,96]. Malfunctions and reduced activity of the transport systems in the compromised RGCs result in the extrusion of excess glutamate, endothelin, ATP, and other cytosolic constituents such as tumor necrosis factor-α [68,86,92,95,97,98]. Extracellular ATP activates purinergic receptors and floods the cells with Ca^2+^, which results in inflammasome-induced cytokine production and release, thereby creating a feed-forward vicious cycle of inflammation, complement activation, induction of autoimmune reactions, and cell death [68,85,86,89,97]. Excess glutamate and endothelin also cause excitotoxicity of the retinal neurons and the optic nerve [99,100], and more damage is inflicted to the retina-optic nerve-thalamic brain neuronal axis. Slowly, the RGC axons retract from the brain neurons, and an atrophic condition prevails, leading to the death of many RGCs. During all these deleterious and stressful events, the patient remains oblivious to the insidious damage being caused by elevated IOP. When the patients have lost over half of the original number of RGCs, they begin to notice diminished peripheral vision, and blind spots appear in their vision. With advancing disease, contrast sensitivity is also reduced [96,100,101,102], and this is the critical time for diagnosis in order to preserve the remaining eyesight. As soon as the ophthalmic examination reveals a decrease in the optic disc area and a corresponding increase in the optic cup and a thinning of the retinal nerve fiber layer (RNFL) [103,104,105] that reflects loss of RGC axons, the clinician has diagnosed glaucomatous optic neuropathy (GON), most likely caused by elevated IOP and/or due to abnormal retinal sensitivity to the prevailing IOP or to the toxins being released due to the local inflammation [63,64,65,68,89,97,106]. Brain defects emerge that increase visual impairment, loss of contrast, blind spots, etc. [107,108,109,110,111,112,113,114]. If the presence of GON is confirmed, the clinician would prescribe eyedrop medication to lower the IOP, perform regular follow-up monitoring of the IOP, and adjust the treatment paradigm as necessary. If the IOP cannot be controlled by one drug and the IOP is reduced to normal levels (14–21 mmHg), adjunctive therapy may be necessary either using single drugs [5,6,7,11,12,14,15,17,20,23,24,25,115,116] or using fixed-dose combination products containing multiple health agency-approved ocular hypotensive drugs [117,118]. If the patient’s IOP is unresponsive to pharmaceutical treatments, then surgery or implantation of tubes or AQH microshunts into the AC of the eye may be necessary to drain the excess AQH [119,120,121,122]. Since this review is focused on pharmaceuticals, only this topic will be pursued further. 

## 3. Major Methods to Lower and Control IOP/OHT

**Figure 4:**
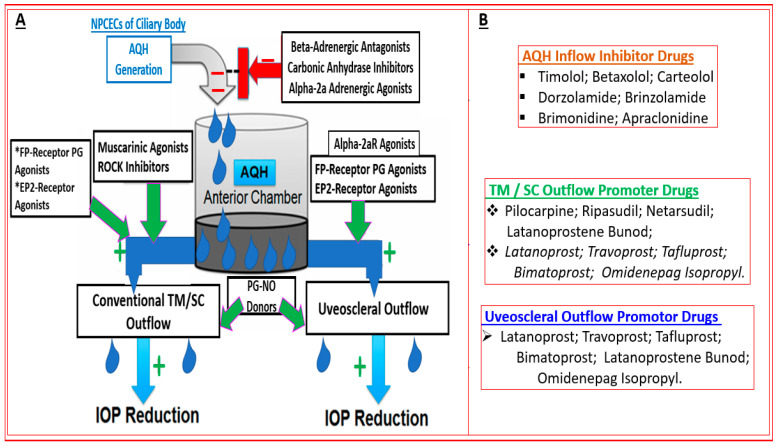
The schematics illustrate the processes of AQH production and its removal from the anterior chamber of the eye via the conventional IOP-dependent outflow pathway (TM/SC) and the uveoscleral pathway. Furthermore, the mechanism of action of key approved medications to lower IOP by influencing the latter process and pathways is depicted in both (**A**) and (**B**). The red minus symbol denotes inhibition and the green plus symbol indicates stimulation or activation.

Three major types of drugs and procedures are utilized today to lower and control IOP. These constitute pharmaceuticals, implantable AQH shunting devices, and surgeries [5,6,7,24,115,119,120,121,122]. Over the years, several classes of compounds have been discovered, tested, pharmacologically characterized, developed into suitable dosage forms, and approved by various world health authorities to impart efficacious ocular hypotensive activity in animal models and glaucoma patients [5,6,7,23,24,25,115,116]. Such pharmaceuticals include miotics (pilocarpine and carbachol; dosed topically ocularly (t.o.) up to four-times daily), beta-adrenergic receptor antagonists (timolol and betaxolol; dosed t.o. twice daily), carbonic anhydrase inhibitors (dorzolamide and brinzolamide; dosed t.o. twice daily), alpha-2 adrenergic receptor agonists (brimonidine and apraclonidine; dosed t.o. once/twice daily), FP-prostaglandin- receptor agonists (latanoprost, travoprost, tafluprost, bimatoprost; dosed t.o. once daily at night), and fixed-dose combination products (dosed t.o. once/twice daily) (Figure 4). Much clinical evidence supports the relatively different magnitudes of IOP-lowering achieved by different classes of these drugs, with FP-receptor agonists having the greatest efficacy and duration of action (25–33% IOP reduced over 24 h), followed by other drugs such as beta-blockers (18–26% IOP reduced), alpha-2-adrenergic agonists (20–25% IOP reduced), muscarinic agonists (20–25% IOP reduced), and carbonic anhydrase inhibitors (15–25% IOP reduced) (Figure 4). These drugs induce a variety of ocular and systemic side effects and, in some cases, have low efficacy or are relatively short-acting. These inadequacies and troublesome off-target activities often lead to patient non-compliance and, in some cases, complete withdrawal from the medications. Due to space limitations and in order to stay focused on the new generation of drugs, the side-effect profiles of the afore-mentioned compounds will not be discussed here but have been reviewed and discussed elsewhere [123,124,125,126,127]. 

During the last several years, new drug classes of compounds with different mechanisms of action from the former drugs have emerged with suitable IOP-lowering and duration of action efficacies in animal models with elevated IOPs see in [7,23,24,80,106,115,127]. Some are undergoing clinical investigations in OHT/OAG patients [115,127]. The other means to lower and control IOP have involved the implantation of miniature devices into the AC using minimally invasive surgeries (MIGS) [120,121]. These AQH microshunts and tubes are manufactured from different biocompatible materials, have different designs and sizes, and have varied locations of AQH drainage from the AC [115,120,121]. Similarly, IOP-lowering has been accomplished using either laser-induced or incisional surgical techniques [115,119,122]. Since all of the latter aspects have been discussed in detail in recent papers and reviews [115,119,121], I shall focus only on the recently approved medications for treating OHT/OAG. 

## 4. Recently Approved Drug-Based Therapeutics for OAG/OHT Treatment

### 4.1. Rho Kinase Inhibitors (Ripasudil and Netarsudil)

**Figure 5:**
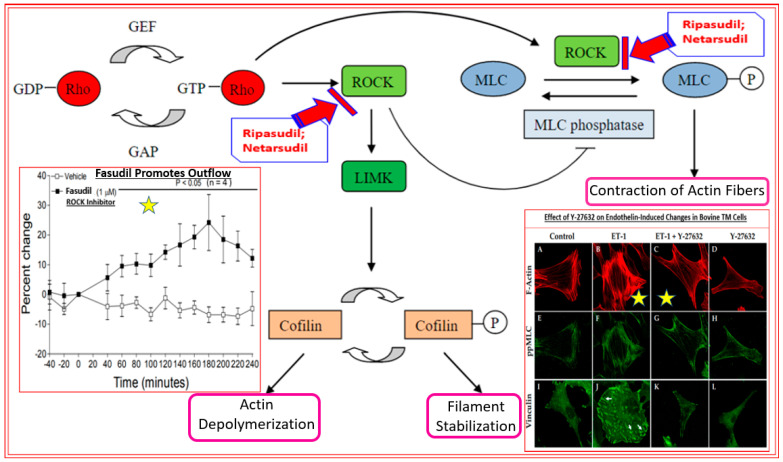
The role of rho kinases in actin fiber contraction, actin depolymerization, and filament stabilization within a TM/SC or CM cell is shown here in a schematic format. The blockage of such processes and events by rho kinase (ROCK) inhibitors such as ripasudil and netarsudil causes the eventual relaxation of the cells. The inset with one yellow star demonstrates the enhancement of fluid outflow from the TM induced by the ROCK inhibitor fasudil in perfused anterior segments of porcine eyes ex vivo. The inset with two yellow stars illustrates the effect of ROCK inhibitor Y-27632 alone or on endothelin-1 (ET-1)-induced contraction of bovine TM cells under in vitro culture conditions, and the changes in F-actin, phosphorylated myosin light chain (ppMLC), and vinculin induced by these compounds.

**Figure 6:**
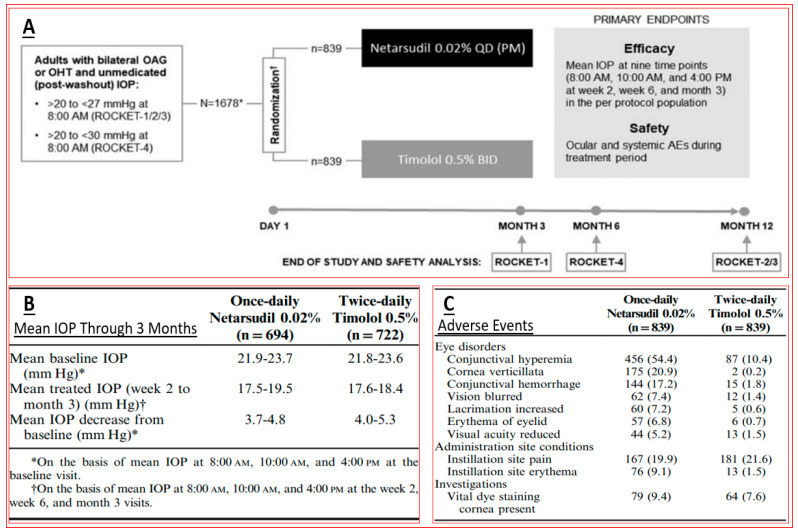
Clinical trial design (**A**), IOP-lowering efficacy data (**B**), and adverse effects (**C**) induced by netarsudil (0.02%, once daily) or timolol (0.05%, twice daily), topically ocularly applied in patients with OAG/OHT are shown.

Until quite recently, the only approved drugs that specifically targeted the conventional outflow pathway of AQH egress from the AC were muscarinic receptor agonists such as pilocarpine and α2-adrenoceptor agonists such as brimonidine, the latter having a relatively small effect (Figure 4; [5,6,7,115,116,117,118,126]). Unfortunately, while these compounds lower IOP reasonably well, their significant side effects severely limit their deployment since the advent and clinical utility of FP-receptor agonist drugs to treat OHT/OAG with less or more tolerable ocular side effects [7,115,116]. Therefore, new TM/SC outflow-promoting therapeutics have been sought as potential replacements for pilocarpine and brimonidine, especially since the use of AQH production inhibitors deprives the tissues lining the AC of nutrients, oxygen, and antimicrobial agents that freshly synthesized AQH brings to the AC. 

Rho kinases 1 and 2 are ubiquitous enzymes found in most mammalian cells. These rho kinases (ROCKs) have a diverse set of roles, amongst which are cell mobility, proliferation, and cell contraction [128,129]. Mammalian ROCK consists of a catalytic enzymic kinase domain at the N-terminus, a coiled-coil region, and a pleckstrin homology domain, which decreases the enzymic activity of ROCKs via an autoinhibitory intramolecular fold if RhoA-GTP is absent. ROCKs promote increases in intracellular Ca^2+^ and induce the formation of stress fibers and focal adhesions by phosphorylating myosin light chain (MLC), which in turn causes actin binding of myosin II, resulting in smooth muscle contraction [128,129,130,131,132,133] (Figure 5). Furthermore, ROCKs are partially responsible for neurite retraction through phosphorylation of collapsin response mediator protein-2 (CRMP2) and inhibiting its role in stimulating axonal outgrowth [128,129] (Figure 5). In the current context of OHT and retinal degeneration, the ROCK enzyme is upregulated in the ONH of glaucomatous eyes [134]. On the positive side, inhibitors of ROCKs promote relaxation of the smooth muscle-like cells within the TM and SC and thereby help AQH exit from the latter tissues, thus lowering IOP. Additional benefits of ROCK inhibitors involve up-regulation and activation of phosphoinositide 3-kinase, Ser and Thr kinase AKT (protein kinase B), and endothelial nitric oxide synthase within the endothelial-like cells of the TM and SC [128,129,130,131]. Such activity results in the liberation of nitric oxide (NO), which relaxes neighboring TM/SC cells to enhance outflow AQH. Much has been written about the beneficial effects of this crosstalk in the context of treating OHT and OAG using either ROCK inhibitors and/or guanylate cyclase activators [53,54] and NO-donors [55,115,116] that generate intracellular cGMP to relax TM/SC tissues [53,54,115,128,129]. Some examples of the first-generation ROCK inhibitors include fasudil, Y-27632 and HA-135 (see, for example, [128,129]; Figure 5, both insets). These compounds exhibited relatively low binding affinity for recombinant human ROCK-I and ROCK-II using a radiotracer assay with inhibitory constants (IC_50_s) in the 1.6–6.7 µM range, which was reflected in their relatively low IOP-lowering efficacies in rabbit and monkey eyes [129,132,133]. Even though the absolute inhibitory constants of many ROCK inhibitors obtained using recombinant human enzymes and a fluorescence polarization assay and the latter assay differ [129], including those values obtained using human TM cells [129], the rank order of potency of the ROCK inhibitors is fairly well reproduced [127,129].

With the afore-mentioned research knowledge, many researchers have successfully created new chemical ROCK inhibitor structures and shown them to impart effective ocular hypotensive activity in animals [128,129,130,131,132,133] and, in some cases, human subjects [135,136,137,138,139,140,141,142,143,144]. A fairly unique property of most, if not all, ROCK inhibitors is that they reduce IOP in both normotensive and OHT eyes [128,129,130,131,132,133]. Ripasudil (Glanatec; IC_50_ = 9 nM) was the first of the ROCK inhibitors to be approved by the health agency in Japan in 2014 for lowering and controlling IOP [135,136]. More recently, the Food and Drug Administration (FDA) granted marketing approval for netarsudil (Rhopressa; IC_50_ = 1 nM) [129,137] in the US for the same indication based on extensive clinical data [138,139,140,141,142,143] (Figure 6). These ROCK inhibitors generally reduce IOP in OHT/OAG patients by 16–20% and 20–25%, respectively, following a single t.o. instilled eyedrop of the medications. As discussed above, ROCK inhibitors stimulate AQH outflow through the conventional TM/SC pathway (Figure 4 and Figure 5). However, due to their vasorelaxant activities, they cause intense hyperemia, conjunctival congestion, corneal verticillata, blurred vision, ocular pain, and general eye irritation (Table 1; Figure 6). A recent report of netarsudil causing punctal stenosis and potentially leading to complete punctal closure also requires attention [140]. In order to enhance the overall ocular hypotensive activity in OAG patients, fixed-dose combinations of netarsudil and latanoprost (e.g., Roclatan; 0.02% netarsudil +0.005% latanoprost) have been achieved, resulting in an overall 31–37% lowering of IOP [118,142]. Such high efficacy has been ascribed to not only stimulation of TM/SC and uveoscleral outflow of AQH, but netarsudil apparently also lowers episcleral venous pressure, and its inhibitory activity at the norepinephrine transporter system probably also contributes to the final IOP reduction observed [137,143]. Even though clinical evidence is currently lacking, in vitro and animal studies have provided data in support of the neuroprotective effects of many different structurally diverse ROCK inhibitors [128,129,144,145,146,147,148,149,150]. If these observations can be replicated in human subjects, ROCK inhibitors have the potential to help directly protect RGCs and perhaps promote neurite extension. Moreover, they may support RGC-axonal elongation and enhance retinal blood flow, potentially offering optic nerve regeneration capabilities in the future [146,147,151]. Such studies are eagerly awaited.

### 4.2. Conjugate of Latanoprost and NO-Donor (Latanoprostene Bunod)

Nitric oxide (NO) is a well-known gaseous transmitter that is produced by NO synthases acting on L-arginine and is released from many different cell types [152,153,154,155,156,157]. NO serves many roles, including relaxation of smooth muscle or smooth muscle-like cells. There are at least three types of NO synthase present in various mammalian cells. These include the calcium-calmodulin controlled isoenzymes eNOS (endothelial NOS) and nNOS (neuronal NOS) [152,153,154,155,156,157]. The inducible isoform, iNOS, also interacts with calmodulin and produces NO for immune defense purposes to eliminate pathogens. Importantly, eNOS has been localized to human TM, SC, and collecting channels, as well as in the ciliary muscle (CM), especially its anterior longitudinal portion [153]. This part of the CM is connected to the TM via tendinous insertions and modulates TM relaxation/contraction to regulate the outflow resistance. Direct t.o. or intracameral application of NO donor agents has been reported to stimulate TM outflow of AQH, and some animal-based and early clinical investigations have indicated that systemic administration of nitro-vasodilators can lower IOP at doses that do not compromise the cardiovascular system [152,154]. A corollary observation to the former situation is that OAG patients responded better to the NO-donor drugs than those with other major types of glaucoma [154,157]. This may be due to the reduced number of eNOS- and iNOS-containing TM and SC cells in OAG patients, and perhaps also due to genetic defects (single nucleotide polymorphisms in eNOS) in the signaling mechanisms associated with the NO-guanylate cyclase coupling system noted in OAG patients [152,153,154,155,156,157]. Thus, it makes sense that exogenously delivered NO-donating compounds would be useful in patients with OHT and/or OAG. Other evidence supports the important role of NO endogenously in affecting TM and SC cellular activities. Thus, human TM cell volume was decreased by NO-independent soluble guanylate cyclase activators YC-1 and BAY-58-2667, which involved the BK_Ca_ ion channel [53], and this is responsible for some of the IOP reduction observed with these types of compounds [116,127,155,156]. Additionally, endogenous regulation of human SC volume was linked to NO signaling in vitro, which directly influences IOP in vivo [54,115,116,127,155,156].

**Figure 7:**
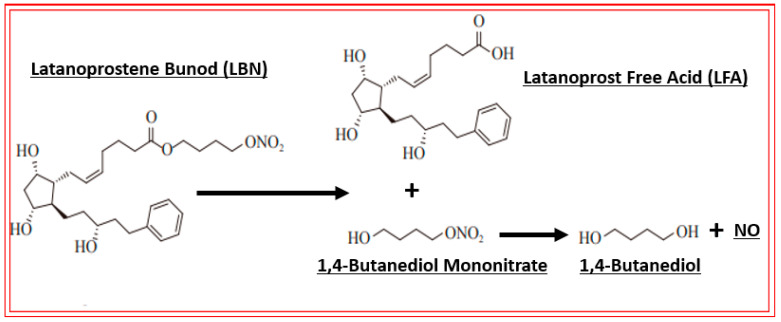
The schematic depicts the chemical structures of latanoprostene bunod (LBN) and the products of its hydrolysis that result in the release of the IOP-lowering FP-receptor agonist latanoprost free acid and the eventual generation of the gaseous transmitter nitric oxide (NO) after topical ocular instillation of LBN. The subsequent interaction of these agents with the FP-receptor and soluble guanylate cyclase, respectively, leads to further downstream signaling events shown in Figure 8A,B.

**Figure 8:**
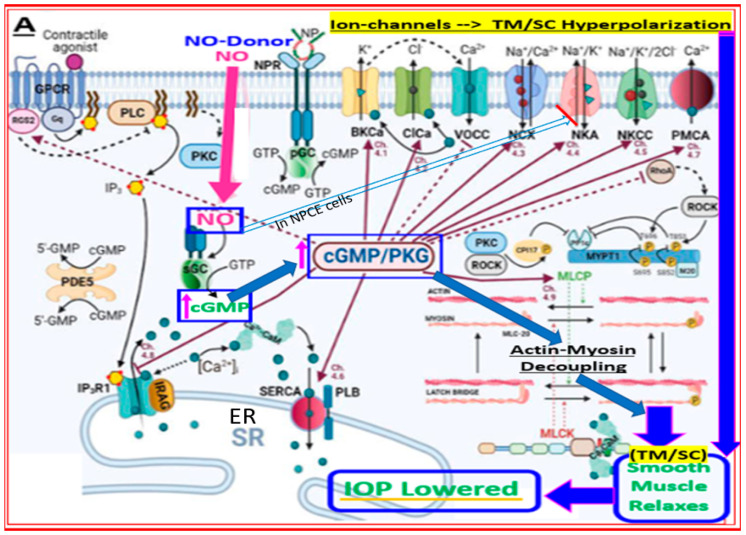

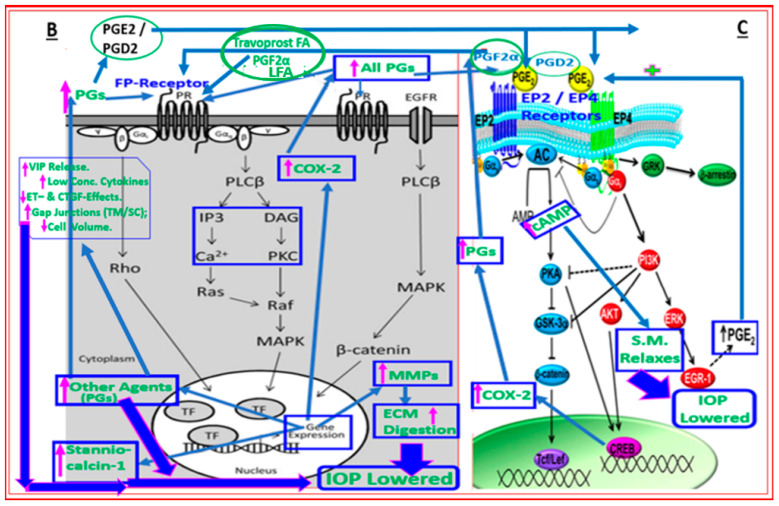
(**A**) illustrates how nitric oxide (NO), either produced by neighboring endothelial-like TM cells or by NO-donor drugs, elicits signaling in the smooth muscle-like TM cells. Binding of NO to soluble guanylate cyclase generates intracellular cGMP, which activates protein kinase G that uncouples actin-myosin to relax the TM cells, which results in enhanced outflow of AQH from the anterior chamber and thus a reduction in IOP. The activated PKG also reduces the availability of intracellular Ca^2+^ by inhibiting the endoplasmic reticular calcium transporter and by inhibiting a range of ion channels that hyperpolarize and relax the TM and /or SC cells to lower IOP. Direct inhibition of rho kinase by PKG is also possible. In the case of non-pigmented ciliary epithelial (NPCE) cells, there is evidence that the NO-cGMP-activated PKG can inhibit Na-K-ATPase to reduce AQH production and help reduce IOP (see the light blue outlined and unfilled arrow). (**B**) shows the various cellular and intracellular components involved in mediating the effects of FP-receptor agonists within smooth muscle cells of the ciliary muscle, TM, and SC to promote efflux of AQH from the anterior chamber of the eye to lower IOP. Here, the phospholipase C activated upon binding of the FP-agonist hydrolyzes cell membrane phospholipids to generate intracellular inositol phosphates and diacyl glycerol, which in turn elicit intracellular Ca^2+^ release and activation of protein kinase C, respectively, leading to enhanced myosin-activated protein kinase (MAPK) activity. Migration and interactions of MAPK with nuclear materials cause the generation and release of pro-matrix metalloproteinases (MMPs) into the cytoplasm of target cells. The latter are cleaved to liberate and activate MMPs that digest ECM to create or enlarge spaces between CM fibers and around TM to allow AQH outflow via both UVS and TM/SC pathways, thereby lowering IOP. Since cyclooxygenase-2 (COX-2) is also increased, additional prostanoids are generated and released into the extracellular space. These bind to their cognate receptors, and the signal transduction is further amplified (e.g. (**B**,**C**)). (**C**). Numerous other events occur, and endogenous hormones/peptides (e.g., Stanniocalcin-1; vasoactive intestinal peptide (VIP)) are released that also help AQH outflow and stimulate additional IOP-lowering.

Due to the existence and success of various fixed-dose combination products used to manage OHT/OAG [117,118,127], and based on the information mentioned above, researchers devised several types of drug conjugates that are held together through hydrolyzable chemical bonds [158]. One such dual-activity conjugate that demonstrated ocular hypotensive activity in animals and human subjects is latanoprostene bunod (LBN; [155,157]; Figure 7 and Figure 8A). As shown below, this combined the FP-receptor agonist functionality with an NO-donating molecule, which, when dosed t.o., penetrates the cornea and is broken down to release latanoprost free acid and NO into the AQH, from where they diffuse to reach several different tissues.

Mechanistically, these individual agents then activate the FP-receptor and guanylate cyclase in their target cells, respectively, to induce the many downstream molecular and cellular events that culminate in promoting AQH efflux from the TM/SC and uveoscleral pathways in order to lower IOP. A major constituent of the TM tissue are smooth muscle-like cells, which rapidly relax and decrease their cellular volume via K^+^-channels in response to the NO-guanylate cyclase-cGMP signaling cascade (Figure 8A; [45,46]). These changes account for the improvement in TM morphology and health, leading to an increase in AQH outflow. Since NO-donor nitro-vasodilator molecules inhibit Na^+^-K^+^-ATPase in the ciliary epithelium [159], additional IOP-lowering by reducing AQH formation may be the outcome. The other major contributor to the IOP-lowering actions of LBN [160] is the FP-receptor agonist, latanoprost free acid (LFA), which is generated after LBN is metabolized down to its constituents (Figure 7). LFA engages phospholipase C upon binding to the FP-receptor and triggers the production of the second messengers, inositol trisphosphates (IP_3_) and diacyl glycerol (DAG), which trigger the release of intracellular Ca^2+^ and activation of protein kinase C, respectively, resulting in cellular contraction [7,20,115,116] (Figure 8B,C). Further amplification of this signaling cascade (Figure 8B,C) occurs via enhancement of gene expression of COX-2 to generate and release endogenous PGs, which can activate EP2 and EP4 receptors to generate cAMP, which causes cellular relaxation. Thus, a series of contraction/relaxation cycles will probably ensue. Additional stimulation of nuclear activity results in the production and release of peptides and hormones such as vasoactive intestinal peptide (VIP) [161] and stanniocalcin-1 [162], respectively. These agents activate their receptors, and other beneficial cellular activities are induced that all culminate in enhancement of AQH outflow via both TM/SC and UVS pathways to lower IOP.

**Figure 9:**
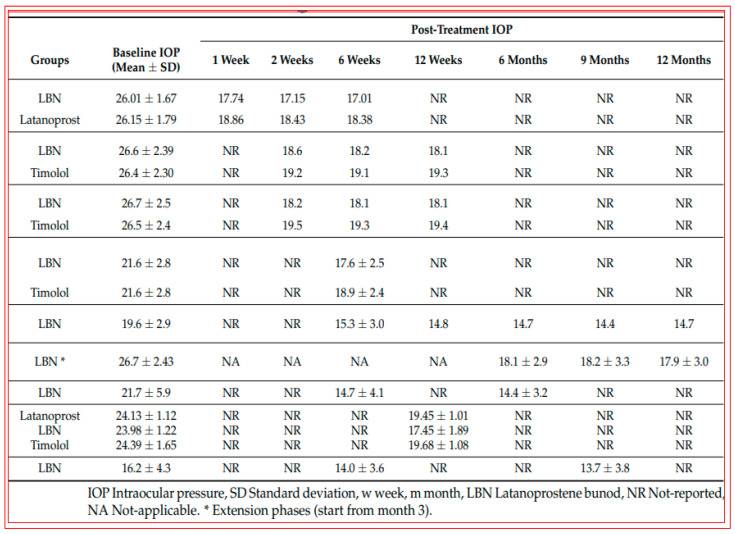
Clinical study data obtained from several investigations of the effect of latanoprostene bunod (LBN; 0.024%, once daily) vs. latanoprost (0.005%, once daily) or timolol (0.05%, twice daily) on IOP changes in OAG / OHT patients covering treatments from 1 week to 12 months are shown.

**Figure 10:**
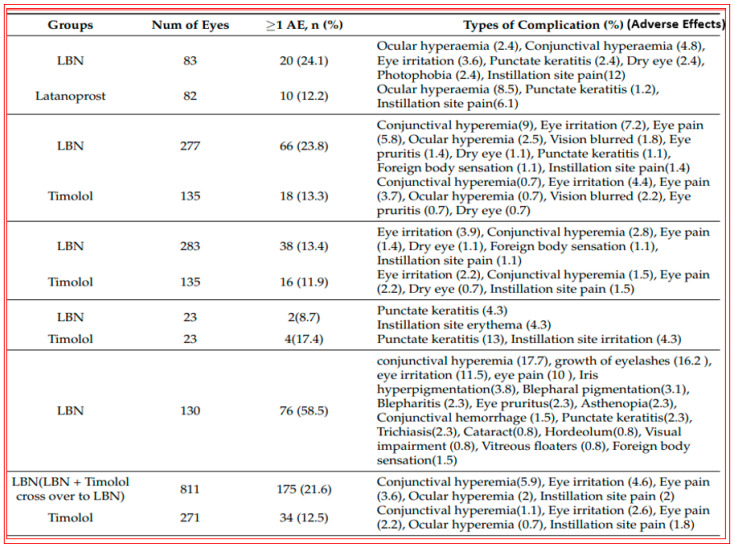
Adverse effects induced by latanoprostene bunod (LBN; 0.024%, once daily), latanoprost (0.005%, once daily), or timolol (0.05%, twice daily) following topical ocular administration in OAG/OHT patients (Figure 9) covering treatments from 1 week to 12 months are shown.

Early animal-based studies demonstrated the good ocular hypotensive efficacy of LBN in normotensive and OHT eyes of rodents (44% drop in IOP 1.5–2 h post t.o. dosing), rabbits and dogs (23–27% drop in IOP 0.5–2 h post t.o. dosing), and Cynomolgus monkeys (31–35% decrease in IOP at 3–4 h post t.o. dosing) [127,155]. In a dose-finding clinical study using varying concentrations of LBN (0.006% to 0.040%) and latanoprost 0.005%, 0.024% LBN was found to be the optimal concentration of LBN, which was then used in phase-III studies [163,164,165,166,167]. OHT/OAG patients tolerated 0.024% LBN well, and IOP was reduced to a greater extent than that induced by 0.05% timolol at all time points, including in the morning. LBN 0.024% lowered IOP by 22% within the first month of t.o. dosing and achieved a 26% reduction by 1 year from baseline, indicating that durable and sustained IOP control was possible with this medication (Figure 9; [163,164,165,166,167]). The side-effect profile of 0.024% LBN was also acceptable (Figure 10 [163,164,165,166,167]). According to all of these characteristics, LBN received marketing authorization from the FDA in 2017 and represents a novel class of conjugated drugs that offer AQH drainage from the AC through both conventional and unconventional pathways. Based on the potential for NO to inhibit Na^+^-K^+^-ATPase, there may be a small contribution to the lowered IOP via inhibition of AQH formation [159].

### 4.3. Sustained Delivery Bimatoprost Implant

Bimatoprost is the amide prodrug of a potent FP-receptor agonist, 17-phenyl PGF_2α_, a free acid. Bimatoprost is metabolized by corneal amidases upon t.o. dosing, and the free acid is considered the active moiety that activates the FP-receptor in the CM and TM to induce IOP-lowering in animals and human subjects [168,169,170,171,172]. As described above for other FP-receptor agonist pro-drugs such as latanoprost and travoprost, bimatoprost free acid binds to the FP-receptor to primarily induce UVS outflow and also increases TM/SC outflow of AQH to reduce IOP (see section under LBN; Figure 8A–C). Whether the recent observation of reduced episcleral venous pressure by bimatoprost in dogs [173] can be reproduced in human OHT/OAG patients remains to be determined. However, given the patient compliance issues described above with t.o. medications for OHT/OAG treatment and to provide sustained delivery of the ocular hypotensive drug over several months, an intracameral injection of a bimatoprost implant was approved by the FDA in 2018 to provide protracted IOP reduction with its attendant side effects [174,175,176,177].

**Figure 11:**
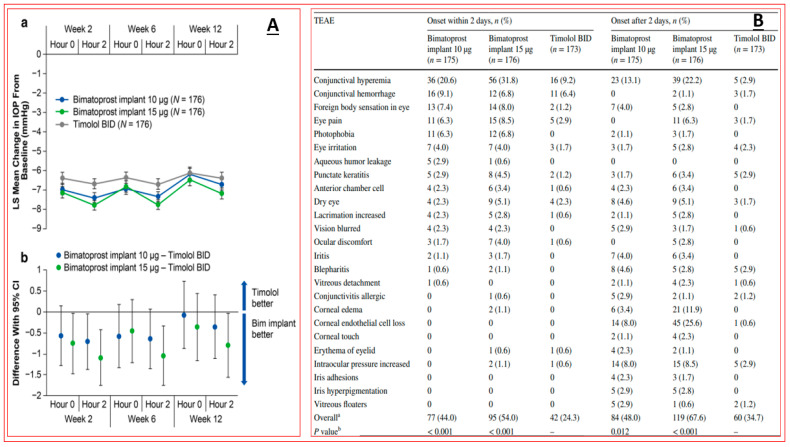
IOP-lowering (**A**; (**a**,**b**)) and adverse effects (**B**) induced by intracameral injection of a sustained delivery polymer rod containing 10 µg or 15 µg of bimatoprost are illustrated. The effect of topically instilled timolol (0.05%, dosed twice daily) on the elicitation of adverse events is also presented for comparison (**B**) within two days and after two days of treatment.

Pooled clinical trial data using 10 or 15 μg of bimatoprost implant revealed at least 20%, 25%, 30%, 35%, and 40% IOP decreases at weeks 2, 6, and 12, which, as expected, were greater than those induced by the comparator timolol [177]; Figure 11. The implant appeared to be well tolerated despite inducing a high level of hyperemia, foreign body sensation, eye pain, photophobia, and overall eye irritation during the first two weeks after implantation. These side effects appeared to subside after this time, but the incidence of corneal endothelial cell loss, blepharitis, corneal edema, iritis, dry eye, and iris pigmentation greatly increased after 2 weeks and appeared to continue increasing over time (Figure 11). Whether physician and patient acceptance of such features of the implant will endure remains to be determined.

### 4.4. Novel Non-Prostaglandin EP2-Prostanoid Receptor Agonist (Omidenepag Isopropyl)

**Figure 12:**
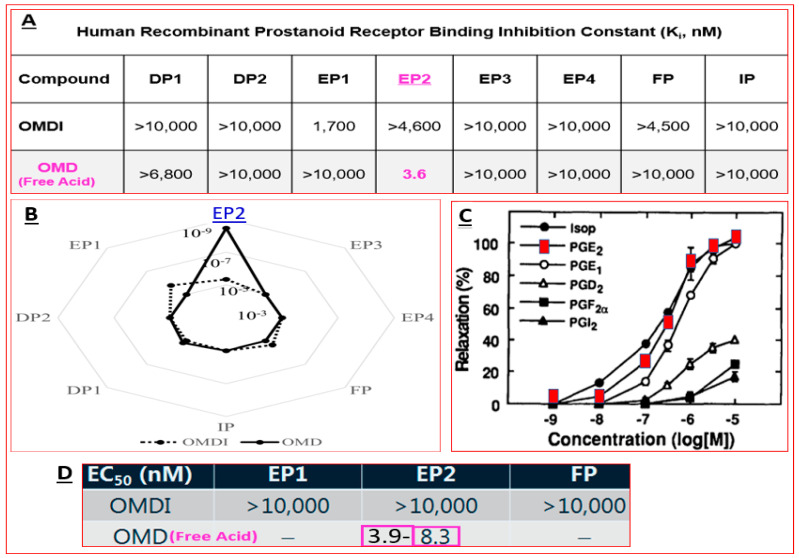
The composite in vitro data pertaining to binding of omidenepag isopropyl (OMDI) and its active free acid (OMD) to various prostanoid receptors and sub-types are shown in (**A**). Since OMD exhibited the highest affinity (lowest binding inhibition constant, K_i_) for the EP2 receptors (**A**) and significantly lower affinities for other receptors, it was deemed to be EP2-receptor-selective compound (**B**). The relaxation of cat ciliary muscle by various prostanoids and isoproterenol in vitro is shown in (**C**). Note the high potency of the natural prostaglandin (PGE2) that interacts mainly with EP2 and EP4 receptors. Finally, the ability of OMD to potently (low nanomolar EC_50_ values) activate adenylate cyclase, only via the EP2-receptor, to generate intracellular cAMP is depicted in (**D**).

**Figure 13:**
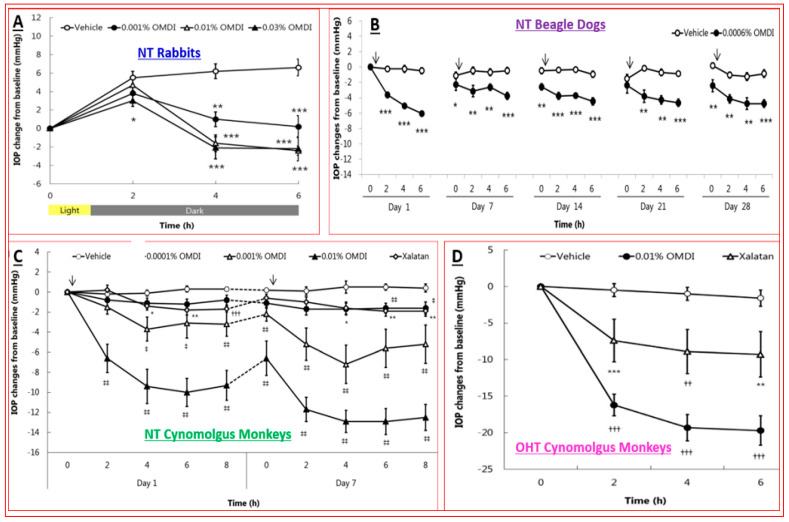
The ability of topical ocularly-instilled omidenepag isopropyl ester (OMDI; various concentrations; once daily), the non-prostanoid EP2-receptor agonist, to lower IOP in ocular normotensive rabbits (**A**), dogs (**B**), Cynomolgus monkey eyes (**C**), and in ocular hypertensive Cynomolgus monkeys (**D**) compared with latanoprost (xalatan; 0.005%) and/or timolol (0.05%) is shown. Note that xalatan did not lower IOP to any appreciable extent in ocular normotensive animals, unlike OMDI. In the ocular normotensive (**C**) and hypertensive monkeys, OMDI (0.001–0.03%) demonstrated either equivalent or greater effectiveness in lowering IOP than latanoprost (xalatan 0.005%). A second dose of OMDI lowered IOP further after the first dose (**C**) without inducing tachyphylaxis of the IOP-reducing effects of OMDI. The statistical significance changes verses controls (baselines) are represented by the asterisks and crosses (*p* < 0.05–0.0001). The arrows signify the t.o. dosing of the compound.

**Figure 14:**
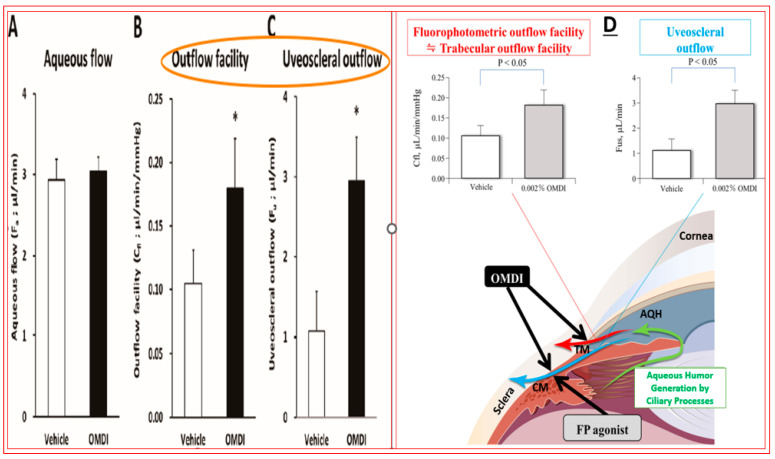
The mechanism of action of OMDI in terms of its ability to influence ocular AQH dynamics was studied in lightly anesthetized ocular hypertensive monkeys following ocular dosing with a single drop of 0.002% OMDI. The figure shows that OMDI promoted AQH outflow via both the conventional TM/SC and the uveoscleral outflow pathways (**B**–**D**) without altering the production of AQH (**A**), unlike FP-receptor agonists such as latanoprost that uniquely and primarily activate the uveoscleral pathway (**D**). Asterisk denotes statistically significant verses the vehicle control.

Over two decades have elapsed since the first prostaglandin (PG) FP-receptor agonist, latanoprost pro-drug, was approved for treating OHT/OAG [116]. During this time, several other PG receptor agonists that stimulated IOP-lowering through other prostanoid receptors reached varying levels of pre-clinical discovery / characterization and clinical development. Some examples include the DP and EP2 receptor agonists (AL-6598); [178,179], EP2-receptor agonists (PF-04217329; ONO-AE1-259-0; Butaprost) [180,181,182], the EP4-receptor agonist [183], the thromboxane receptor agonist [184], and the dual FP/EP3 receptor agonist (ONO-9054; Sepetaprost; [185]) with ocular hypotensive activities. The most advanced and recently approved EP2-receptor agonist, which is a unique non-prostanoid drug, is omidenepag isopropyl ester (OMDI; 0.002%), which was approved and marketed in Japan in 2018 as Eybelis and approved by the FDA in 2022 and marketed as Omlonti for lowering and controlling IOP [115,116,186,187,188]. Omidenepag is the free acid active moiety released into the AQH following hydrolysis of OMDI by corneal esterases after t.o. dosing of OMDI [187]. OMD exhibits a high affinity and selectivity for the EP2-receptor and behaves as a full agonist to induce cAMP production within the target cells of tissues of the outflow (TM/SC; CM/sclera) and inflow (ciliary epithelium) pathways [187,188]; Figure 12A,D. Amongst many drugs and drug candidates, 0.002% and 0.01% OMDI exhibited one of the fastest onsets of action in terms of reducing IOP in many mammalian species, including conscious and sedated Cynomolgus monkey eyes, inducing 44–56% IOP reduction in the latter between 1–4 hrs post t.o. dosing [127,187,188] (Figure 13A–D). The mechanism of action in the mildly sedated monkey eyes was shown to be activation of mainly the UVS outflow of AQH, with an additional contribution from the stimulation of the TM/SC outflow pathway [188], Figure 14. A possible influence of OMD on the AQH inflow mechanism cannot be fully ruled out since functional EP2 receptors are also present on non-pigmented ciliary epithelial cells [189,190] in addition to those found on the CM, TM, and SC [191]. Due to the involvement and ability of cAMP produced by EP2 receptor activation by OMD and the potential up-regulation of inducible cyclooxygenase-2 [192] to stimulate production of endogenous PGs [193,194,195], it is likely that a combination of smooth muscle relaxation (ciliary muscle, TM, and perhaps Schlemm’s Canal (SC) cells) [196,197,198,199] (Figure 4 and Figure 8C) and MMPs-induced reduction in ECM in and around TM/ ciliary muscle fibers/sclera [191,200] is responsible for the high IOP-lowering efficacy of OMDI (Figure 13). OMD also directly suppressed mRNAs and proteins associated with tissue growth factor β2 (TGFβ2)-induced ECM deposition [201], and downregulated mRNAs for fibronectin1, collagen-12A1 and collagen-13A1 in 2D-cultures of human TM cells, thereby reducing the ECM components that pathologically cause outflow resistance [202]. Relaxation of the smooth muscle cells associated with ciliary muscle, TM, and SC cells by EP2 agonists (Figure 12C) [196,197,198,199] also results in enhanced AQH drainage and ocular hypotension [180,181,188,202]. Taken together, the active forms of EP2-receptor agonists, including OMD, appear to powerfully reduce and control IOP in a sustained manner in multiple species (Figure 13A–D) [115,187]. They accomplish this by recruiting several molecular and cellular components and factors within the eye’s anterior chamber tissues (Figure 4 and Figure 8C). Moreover, it seems that EP2 receptor agonists can also provide protection against endoplasmic reticulum stress in TM cells [203] and reduce the potential conversion of smooth muscle TM cells to a fibroblastic phenotype [204], thereby preserving the integrity and functionality of the AQH drainage system. The multiplicity of actions of OMD observed in vitro and in vivo in animal models of OHT bode well for potentially high ocular hypotensive efficacy in human subjects.

**Figure 15:**
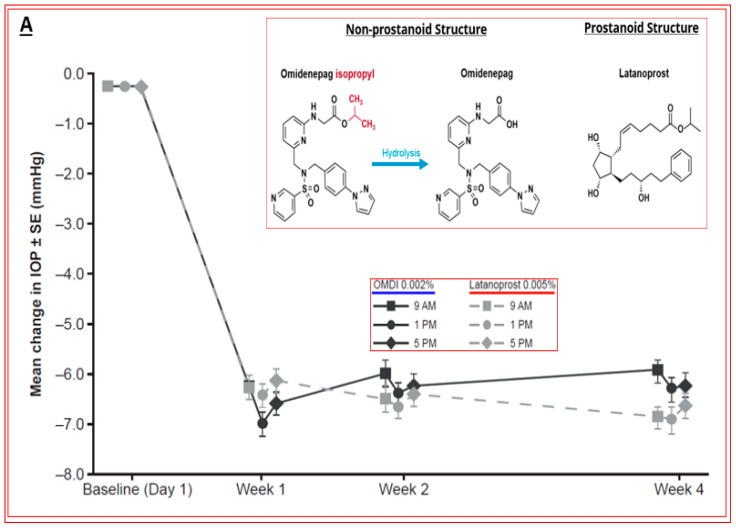

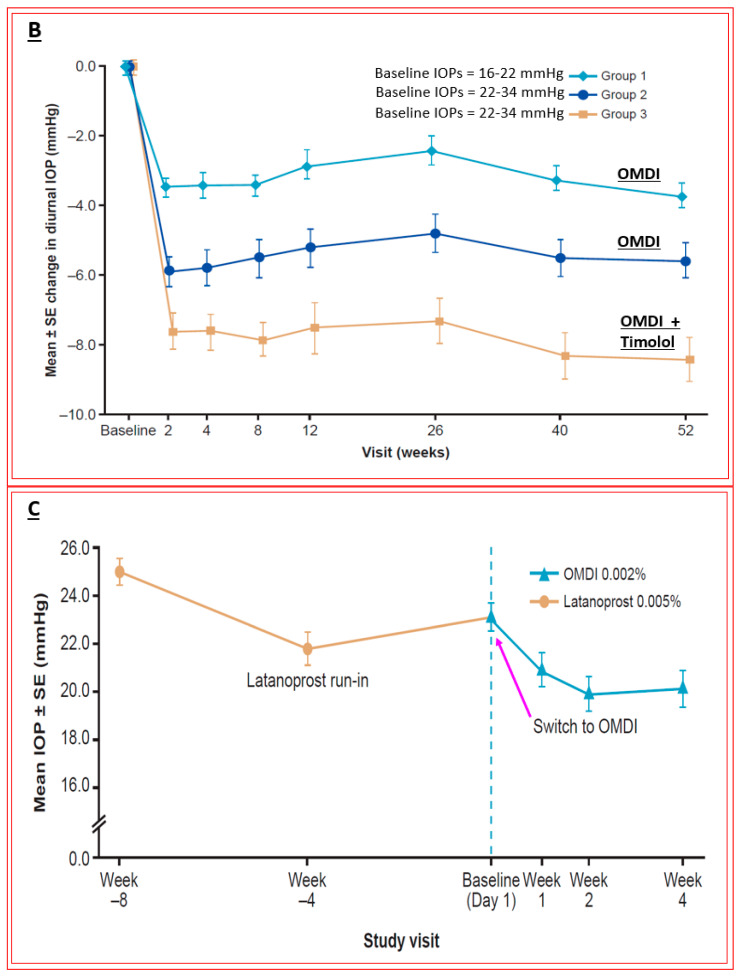
(**A**). Clinical study data comparing the IOP-lowering efficacy of topical ocularly administered OMDI (0.002%; once daily) and latanoprost (0.005%; once daily) in patients with OHT/OAG is shown for treatment spanning 1–4 weeks (**A**). A closely similar time-course of efficacy is evident between the two different classes of drugs (**A**). In the inset are shown the chemical structures of OMDI and its free acid (OMD), which have a non-prostanoid structure and bind to EP2 receptors, as compared to latanoprost, which has typical prostaglandin structural features and whose free acid interacts with the FP-receptor. (**B**) displays the clinical efficacy of OMDI (0.002%, once daily) in lowering and controlling IOP over 1 year when tested alone in OHT/OAG patients with low baseline IOPs (Group 1; ocular normotensives) or in patients with high baseline IOPs (Group 2; ocular hypertensives), and when tested in the latter group of patients in conjunction with timolol (0.05%, twice daily dosing) (Group 3; ocular hypertensives). (**C**) depicts the change in IOP reduction when OHT/OAG patients who were low responders to latanoprost (0.005%, once daily dosing) were switched over to topical ocular treatment with OMDI (0.002%, once daily dosing) over a 4- week period of study.

Phase-I clinical studies and the respective OMDI pharmacokinetics data indicated a promising safety and tolerability profile and demonstrated no accumulation following 7 days of t.o. dosing and a short half-life in plasma indicative of low systemic exposure [205]. Taken together, three dose-finding studies and a dose-frequency study demonstrated that, of the seven doses of OMDI studied, 0.002% and 0.0025% induced the best IOP-reducing actions, which were similar to those of latanoprost 0.005%. Since OAG and other forms of glaucoma are chronic diseases requiring long-term t.o. dosing to control IOP, the lowest effective dose is preferred to obtain the highest therapeutic index. Thus, 0.002% OMDI was considered the optimal concentration to achieve the desired IOP reduction with a favorable side-effect profile and ocular hypotensive efficacy comparable to latanoprost 0.005% [206]. In phase-III clinical trials of OMDI ophthalmic solution 0.002% (Figure 15A–C), two studies used patients with glaucoma or OHT (SPECTRUM 3, NCT03691649; and SPECTRUM 4, NCT03691662); two investigations enrolled patients with open-angle glaucoma or OHT (RENGE, NCT02822729; and PEONY, NCT02981446) [207]; one trial comprised a OAG or OHT patient population (AYAME, NCT02623738) [208]; and two trials used patients with OAG or OHT who were non-/poor responders to latanoprost 0.005% (FUJI, NCT02822742; and SPECTRUM 5, NCT03697811) [209]. The clinically beneficial efficacy of OMDI in treating patients with OAG or OHT was confirmed in phase-III trials that demonstrated IOP-lowering non-inferiority to those with latanoprost 0.005% (AYAME; PEONY) or those with timolol 0.5% (SPECTRUM 4; NCT03691662) and sustained IOP reduction alone (SPECTRUM 3; RENGE) or in combination with timolol 0.5% (RENGE) (Figure 15A,B).

OMDI as a treatment option for non-/low responders to latanoprost was established in an open-label phase-III investigation (FUJI; NCT02822742) that demonstrated an additional ~3 mmHg IOP-lowering in patients after switching to OMDI for 4 weeks from latanoprost 0.005% (Figure 15C). This additional IOP-reducing action observed with OMDI has also been demonstrated in US patients in the phase-III SPECTRUM 5 trial, with reductions in IOP of the same order as those in Japanese patients in the FUJI study [210].

The SPECTRUM 5 clinical study was an open-label, multicenter study in patients with OAG or OHT who were latanoprost low-/non-responders, i.e., those that did not achieve a ≥15% reduction in IOP from the end of the washout period to the end of the latanoprost run-in period. A total of 107 patients were enrolled and were t.o. treated with OMDI 0.002% once daily in the evening for 3 months, and their IOPs were measured at three timepoints (08:00, 12:00, and 16:00) on their scheduled visits at Week 2, Week 6, and Month 3. The primary efficacy endpoint of this trial was met (i.e., significant change from baseline in mean diurnal IOP at Month 3). OMDI 0.002% t.o. once daily resulted in an early onset of IOP reduction at the first post-baseline visit (Week 2) and a continued, stable IOP-reducing activity throughout the remainder of the trial. OMDI 0.002% was well tolerated; no patients discontinued due to AEs, and no safety concerns of significance were reported. Of the nine cases of conjunctival hyperemia, eight were mild in severity, and only one case of an increase in eyelash pigmentation was noted.

**Figure 16:**
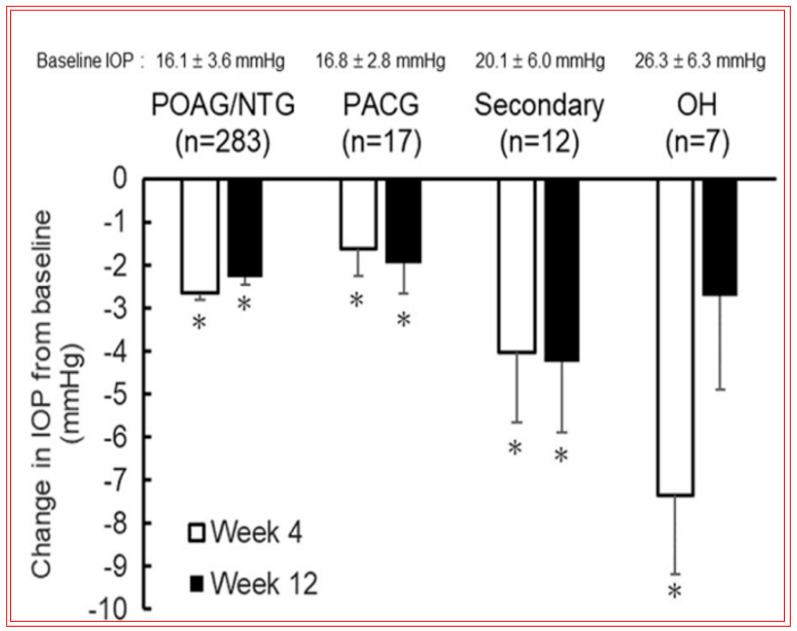
Results from clinical evaluation of OMDI (0.002%; once daily topical ocular dosing) in a variety of patients afflicted with different types of glaucomas are displayed. The data show that OMDI exhibited different levels of ocular hypotensive activity in these varied populations of glaucoma patients. Apparently, OMDI lowered the highest magnitude of IOP in patients with OHT and secondary forms of glaucoma and exerted slightly lower efficacy in a group of OAG/NTG and ACG patients. Nevertheless, OMDI is an effective ocular hypotensive medication that is well tolerated and is effective in all four types of glaucoma/OHT patients after 4 and 12 weeks of treatment. Asterisk denotes statistically significant verses the baseline values.

The novel mechanism of action of OMDI (IOP reduction by recruiting both the conventional and uveoscleral outflow pathways) may explain the additional IOP-reducing effect in both the FUJI study in Japanese patients and the SPECTRUM 5 trial in the US-based patient population. Of note was that results from the RENGE trial showed IOP reductions with OMDI of 0.002% achieved in patients with normal IOPs (i.e., baseline IOPs were ≥16 < 22 mmHg), suggesting that OMDI may be effective in patients with normal tension glaucoma (NTG) (Figure 15B) [211]. Clearly, the higher the baseline IOPs, the greater the efficacy of OMDI 0.002% (Figure 15B). More recent studies indicated that OMDI may also be therapeutically useful in Japanese patients with secondary glaucomas and primary angle-closure glaucoma (Figure 16; [211,212,213]). This is where a relatively fast onset of action of OMDI may be beneficial, as identified from the monkey studies [127,187,188]. However, these results require confirmation via additional clinical investigations in Japanese patients and in patients of different ethnicities in order to support these initial observations. Nevertheless, OMDI can be combined with other ocular hypotensive drugs approved for treating OHT/OAG. Data from monkey and human studies provide support for such adjunctive therapies [187,207,214] (Figure 13 and Figure 15B).

**Figure 17:**
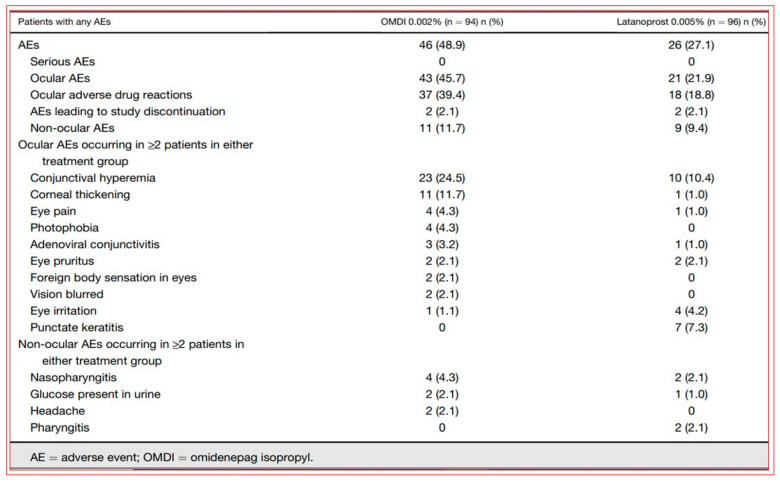
Adverse events observed with topical ocular instillation of omidenepag isopropyl (OMDI; 0.002%, dosed once daily) or latanoprost (0.005%, once daily dosing) in OHT/OAG patients in a phase-III study are shown for comparison.

**Figure 18:**
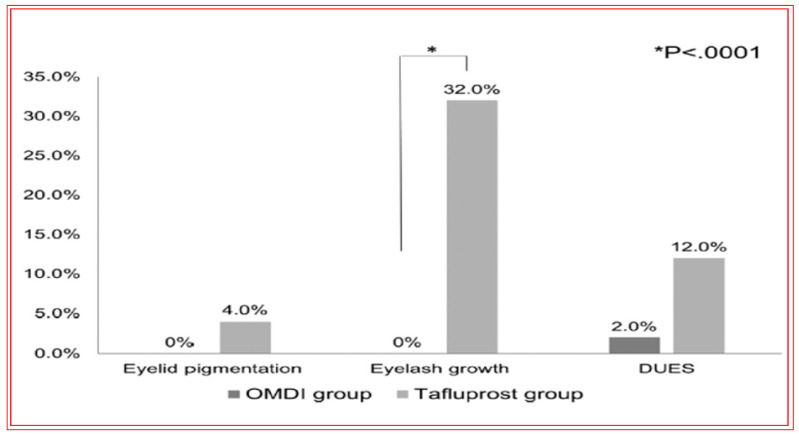
The objective symptom evaluations of periocular adverse reactions in patients receiving topical ocular OMDI (0.002%, once daily) or tafluprost (an FP-receptor agonist; once daily dosing) are shown. Comparative data pertaining to measures of eyelid pigmentation, eyelash growth, and deepening of the upper eyelid sulcus (DUES) are illustrated.

**Figure 19:**
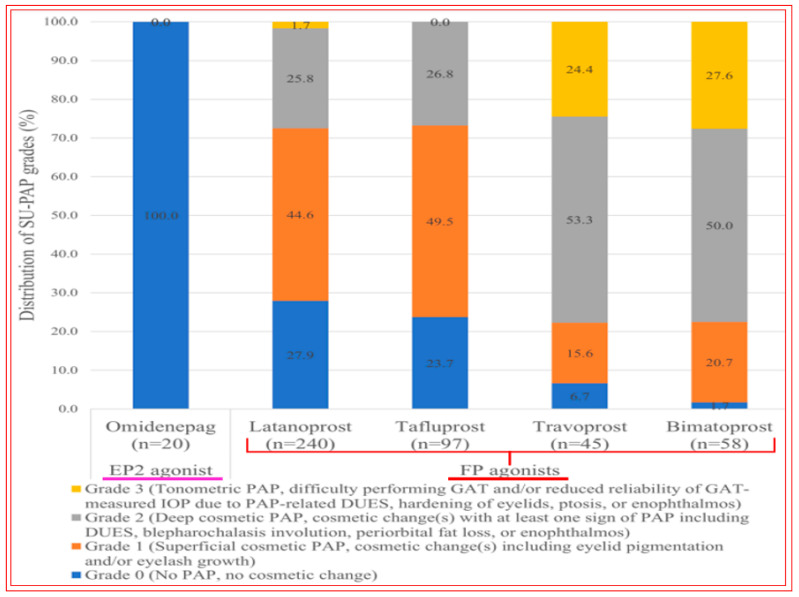
The relative propensity to induce prostanoid-associated periorbitopathy (PAP) symptoms or other ocular and adjacent tissue cosmetic alterations by four different FP-receptor agonists and an EP2-receptor agonist, OMDI, following topical ocular treatment in ocular hypertensive or glaucoma patients is displayed. The overall severity of the changes induced is shown on a relative scale or graded format ranging from Grade 0 to Grade 3.

Of the ocular side effects with OMDI (in the AYAME and FUJI trials), conjunctival hyperemia was the most common, although this was rather mild in the majority of the subjects. Corneal thickening was also noted (Figure 17). Mild or moderate macular edema/cystoid macular edema was noted in 14 subjects, but all of these happened in pseudophakic patients in the RENGE trial (Figure 17). These results support the contraindication of OMDI use in pseudophakic patients as noted in the OMDI 0.002% approved drug label in Japan. Prostaglandin-associated periorbitopathy (PAP) eyelid pigmentation, eyelash growth, and DUES have been reported for all t.o. used FP-receptor agonist drugs [116,124,125,126,215,216] (Figure 18). However, in comparison with the latter class of drugs, including bimatoprost and travoprost, OMDI 0.002% induced a much lower frequency (0–2%) of PAP symptoms [217,218,219] (Figure 18 and Figure 19). These data suggest that OHT/OAG patients may benefit from switching to treatment with OMDI 0.002% rather than continuing with FP-receptor agonists and being susceptible to the associated appearance-altering AEs. However, the t.o. treatment paradigm for OHT/OAG patients is in the domain of the treating clinician. The RENGE trial data indicated no appearance-altering side effects throughout the year-long trial. Other researchers have also reported similar results when studying the appearance-altering side effects of t.o. OMDI 0.002% or tafluprost in patients with newly diagnosed OAG or OHT (Figure 18). Thus, in comparison with tafluprost-treated eyes, those that received t.o. OMDI 0.002% exhibited significantly fewer cases of eyelash growth (0% vs. 32%), a lower rate of eyelid pigmentation (0% vs. 4%), and a lower incidence of DUES (2% vs. 12%). Additional results from the deployment of OMDI 0.002% in a post-marketing real-world study of >1800 Japanese patients reported IOP reductions in patients with normal IOP (≤16 mmHg), consistent with the findings of the RENGE trial in patients with NTG [212]. Furthermore, this new study described no cases of PAP symptoms, cystoid macular edema, or uveitis, further strengthening the safety profile of OMDI 0.002%. Therefore, the clinical evidence gathered thus far suggests that OMDI 0.002% is non-inferior to first-line FP-receptor agonists such as latanoprost 0.005% and is a suitable alternative to the latter class of t.o. medications, thereby avoiding the undesirable facial appearance-altering side effects linked to the use of FP-receptor agonists. Various animal-based and clinical studies have also demonstrated that OMDI can be combined with other IOP-lowering drugs to obtain additional efficacy [187,207,208,214] (Figure 13 and Figure 15B). Such adjunctive therapy will prove useful for patients with many types of glaucoma [212]. Furthermore, recent clinical studies involving OMDI 0.002% demonstrated its beneficial ocular hypotensive properties in patients suffering from not only OHT and OAG, but it also lowered IOP significantly in NTG and ACG patients as well over a 4- week trial period (Figure 16) [127,212,213].

## 5. Conclusions

The available care and long-term management of glaucoma vary, but the visual disability is mainly preventable when detected and treated in time. Early diagnosis remains a huge challenge. Nevertheless, significant progress has been made in better understanding the pathological aspects of and combating OHT and OAG over the last 2–3 decades [5,6,7,8,9,10,220,221]. The major contributor to this has been the advent and acceptance of a key modifiable biomarker that triggers appropriate treatment initiation, namely elevated IOP [11,12,13,14,15,16,17,18,19,20,21,22,70,71,72,73,74,75,76,104,105]. With this came the creation, validation, and deployment of numerous types of animal models of OHT (natural and induced) [7,126,127,222,223,224,225,226] to permit screening of potential ocular hypotensive drug candidates. While translation of many such agents has lagged behind, a number of new clinically viable drugs with high enough therapeutic indices have emerged to which patients have responded with acceptable tolerability and efficacy. Three major classes of recently approved and marketed pharmaceuticals have thus been discussed in this review, viz., rho kinase inhibitors (ripasudil (Glanatac^®^) [135,136]; netarsudil (Rhopressa^®^)) [137,138,139,140], a conjugate of an FP-receptor agonist and an NO-donor (latanoprostene bunod (Vyzulta^®^)) [155,156,157,158,159,160,161,162,163,164,165,166,167], and non-prostanoid EP2-receptor agonist (omidenepag isopropyl (Eybelis^®^, Omlonti^®^)) [186,187,188,201,202,203,204,205,206,207,208,209,210,211,212,213,214,215,216,217,218,219]. In order to overcome compliance issues, a sustained delivery platform was used to create an intracameral injectable polymer-based rod containing an old pro-drug FP-receptor agonist, bimatoprost (Durysta^®^) [174,175,176].

On the horizon are a number of pharmaceutical agents in clinical trials that are embedded in sustained-release vehicles, including a silicone ring containing bimatoprost, punctal plugs, and contact lenses that release travoprost or latanoprost, and a microshunt that would release travoprost [220]. A novel dual-activity compound expressing FP- and EP3-receptor agonist properties that lowers IOP in animals and human subjects, sepetaprost, has advanced to late-stage clinical evaluation [185]. New classes of compounds with adequate IOP-lowering activity and side-effect profiles in animals are also emerging but require investigative new drug-enabling studies or further phases of drug development to permit their evaluation for durable clinical acceptability as new ocular hypotensive drugs [7,115,116,127,128,129,227,228]. A few examples include an anticonvulsant, analgesic, and anxiolytic medication used to treat epilepsy, neuropathic pain, fibromyalgia, restless leg syndrome, opioid withdrawal, and generalized anxiety disorder that has been repurposed as an IOP-reducing agent (pregabalin [228]), a prostaglandin EP2-receptor agonist [227], an autotaxin/lysophosphatidic acid receptor antagonist [229] and a TRPV-4 antagonist [50,51] (see [127] for more information on these and additional investigative drug candidates).

In addition to the traditional small-molecule drugs to treat OHT/OAG discussed above, new techniques and technologies are emerging that could potentially provide durable IOP reduction without needing medication eyedrops. These include gene-directed therapies involving the use of small interfering ribonucleic acids (siRNAs) that target relevant receptors (e.g., beta-adrenergic [230]), blocking actions of cytokines [231,232], gene-therapy that introduces RNAs for MMPs to clear ECM around the TM cells [233], and the use of gene-editing technology (CRISPR-Cas9) to affect myocilin [234] or aquaporin-channels to modulate AQH dynamics [235].

In view of the fact that many patients with NTG and/or OAG/OHT on maximally accepted therapy still continue to progress towards more severe visual disability. It indicates that IOP is not the only factor that needs to be modified to claim success in managing the degenerative ocular diseases [5,6,7,10,11,12]. Consequently, direct protection of RGCs, the RGC axons, the optic nerve, and brain thalamic and visual cortical neurons is necessary in addition to decreasing and controlling IOP [236,237,238,239,240]. Consequently, many approaches in this realm are at various stages of maturity in terms of demonstrating neuroprotective and/or regenerative activities. Moreover, potentially disruptive long-term treatment paradigms for OHT/OAG and other forms of glaucoma encompass cell-replacement therapies for both the anterior chamber tissues such as the TM [241,242,243] and also for RGCs in the retina [241,244]. Different types of electrical, electromagnetic, ultrasonic, and optogenetic technologies are also emerging that would be of benefit to patients suffering from OHT/OAG and other glaucomatous diseases [245,246,247]. Many aspects of these novel therapeutic modalities have been recently reviewed and discussed and cover the potential therapeutic effects of AQH-derived and/or retinal organoid-derived extracellular vesicles and their cargos, such as miRNAs and trophic factors, and even the use of nutraceutical approaches to curb vision loss [236,237,238,239,240,241,242,243,244,245,246,247,248,249,250]. Likewise, to aid in the early detection and diagnosis of OHT, OAG, and other degenerative retinal and brain diseases, several novel tools are being evaluated and have advanced to different degrees of predictability, reliability, and acceptability by the scientific community [251,252,253,254,255,256]. We hope that many of the latter diagnostic technologies and therapeutic modalities will enter clinical trials and proceed towards health authority approvals in the near future. Such progress should offer more hope for patients suffering from the afore-mentioned ocular degenerative disorders. Researchers in academia and the biopharmaceutical industry need to keep these patient- and people-centric issues in mind as they forge the pathways for discovering and bringing forward new treatments to help these patients with ocular afflictions such as OHT, OAG, and other glaucomas.

## Figures and Tables

**Table 1:** Recently approved Eyedrop Medications for Treating OHT and OAG.

Eyedrop Medication and Year of Health Agency Approval	Common Name and Pharmacological Type of the Drug. (IOP Reduction Achieved in OHT/OAG Patients)	Topical Ocular Dosage Concentration and Form (%, *w*/*v*)	Topical Ocular Dosing Frequency	Mode(s) of Action to Reduce IOP	Common Adverse Effects
**Glanatec (2014 Japan)** 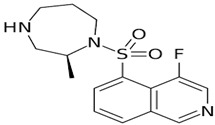	Ripasudil (Rho kinase (ROCK) inhibitor) (3.5–4.5 mmHg IOP reduction; 16–20% reduction)	0.4% solution	1 drop twice daily	Enhances conventional (TM) outflow of AQH	Conjunctival congestion, conjunctival inflammation, blepharitis and eye irritation.
**Rhopressa (2017)** 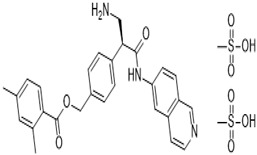	Netarsudil (Rho kinase (ROCK) inhibitor) (5 mmHg IOP reduction; 20–25% IOP decrease)	0.02% solution	1 drop daily at night	Enhances conventional (TM) outflow of AQH; also decreases episcleral veinous pressure	Conjunctival hyperemia, corneal verticillata, blurred vision, instillation site pain, and conjunctival hemorrhage.
**Vyzulta (2017)** 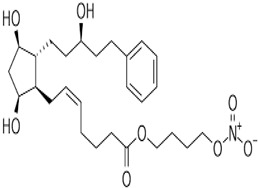	Latanoprostene Bunod (conjugate of latanoprost and an NO-donor agent); (32–34% IOP reduction)	0.024% solution	1 drop at night	Enhances AQH outflow via the UVS pathway and via TM/SC pathway	Eye discomfort/irritation, hyperemia, temporary blurred vision, increase in eyelash number/length/thickness and darkening of the eyelashes /eyelids and iris.
**Durysta Implant (2020)** 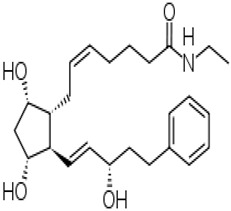	Intracamerally injected sustained-delivery biodegradable polymer containing bimatoprost (7.5 mmHg IOP reduction)	Not applicable	Once implanted into the ANC of the eye (intracameral injection), the drug elutes off the implant over 6 months.	Enhances AQH outflow via the UVS pathway and via TM/SC pathway	Conjunctival hyperemia, foreign body sensation, eye pain, photophobia, conjunctival hemorrhage dry eye, eye irritation increased IOP, corneal endothelial cell loss, vision blurred, iritis, headache.
**Eybelis (2018 Japan)** Omlonti (2022 USA) 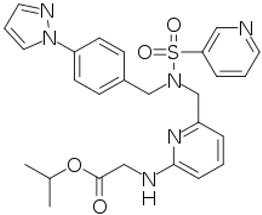	Omidenepag Isopropyl (Non-prostaglandin EP2-receptor selective agonist); (28.6–29.2% IOP reduction)	0.002% solution	1 drop daily at night	Enhances AQH outflow via the UVS pathway and via TM/SC pathway	Transient conjunctival hyperemia, corneal thickening.

## Data Availability

Data sharing is not applicable.

## References

[1] WHO Blindness and Vision Impairment. Fact Sheets. WHO Priority Eye Diseases. https://www.who.int/blindness/causes/priority/en/.

[2] Burton M.J., Ramke J., Marques A.P., Bourne R.R.A., Congdon N., Jones I., Ah Tong B.A.M., Arunga S., Bachani D., Bascaran C. (2020). The Lancet global health commission on global eye health: Vision beyond. Lancet Glob. Health.

[3] Sun Y., Chen A., Zou M., Zhang Y., Jin L., Li Y., Zheng D., Jin G., Congdon N. (2022). Time trends, associations and prevalence of blindness and vision loss due to glaucoma: An analysis of observational data from the Global Burden of Disease Study 2017. BMJ Open.

[4] Tham Y.C., Li X., Wong T.Y., Quigley H.A., Aung T., Cheng C.Y. (2014). Global prevalence of glaucoma and projections of glaucoma burden through 2040: A systematic review and meta-analysis. Ophthalmology.

[5] Weinreb R.N., Aung T., Medeiros F.A. (2014). The pathophysiology and treatment of glaucoma: A review. JAMA.

[6] Jonas J.B., Aung T., Bourne R.R., Bron A.M., Ritch R., Panda-Jonas S. (2017). Glaucoma. Lancet.

[7] Sharif N.A. (2021). Therapeutic Drugs and Devices for Tackling Ocular Hypertension and Glaucoma, and Need for Neuroprotection and Cytoprotective Therapies. Front. Pharmacol..

[8] (2021). European Glaucoma Society terminology and guidelines for glaucoma. Br. J. Ophthalmol..

[9] Chan P.P., Pang J.C., Tham C.C. (2019). Acute primary angle closure-treatment strategies, evidence and economic considerations. Eye.

[10] Mallick J., Devi L., Malik P.K., Mallick J. (2016). Update on normal tension glaucoma. J. Ophthalmic Vis. Res..

[11] Collaborative Normal-Tension Glaucoma Study Group (1998). Comparison of glaucomatous progression between untreated patients with normal-tension glaucoma and patients with therapeutically reduced intraocular pressures. Am. J. Ophthalmol..

[12] Collaborative Normal-Tension Glaucoma Study Group (1998). The effectiveness of intraocular pressure reduction in the treatment of normal-tension glaucoma. Am. J. Ophthalmol..

[13] (2000). AGIS Investigators The Advanced Glaucoma Intervention Study (AGIS): The relationship between control of intraocular pressure and visual field deterioration. Am. J. Ophthalmol..

[14] Kass M.A., Heuer D.K., Higginbotham E.J., Johnson C.A., Keltner J.L., Miller J.P., Parrish R.K., Wilson M.R., Gordon M.O. (2002). The Ocular Hypertension Treatment Study: A randomized trial determines that topical ocular hypotensive medication delays or prevents the onset of primary open-angle glaucoma. Arch. Ophthalmol..

[15] Tezel G., Siegmund K.D., Trinkaus K., Wax M.B., Kass M.A., Kolker A.E. (2001). Clinical factors associated with progression of glaucomatous optic disc damage in treated patients. Arch. Ophthalmol..

[16] Gordon M.O., Beiser J.A., Brandt J.D., Heuer D.K., Higginbotham E.J., Johnson C.A., Keltner J.L., Miller J.P., Parrish R.K., Wilson M.R. (2002). The Ocular Hypertension Treatment Study: Baseline factors that predict the onset of primary open-angle glaucoma. Arch. Ophthalmol..

[17] Heijl A., Leske M.C., Bengtsson B., Hyman L., Bengtsson B., Hussein M., Early Manifest Glaucoma Trial Group (2002). Reduction of intraocular pressure and glaucoma progression: Results from the Early Manifest Glaucoma Trial. Arch. Ophthalmol..

[18] Leske M.C., Heijl A., Hyman L., Bengtsson B., Komaroff E. (2007). Predictors of long-term progression in the early manifest glaucoma trial. Ophthalmology.

[19] Bengtsson B., Leske M.C., Hyman L., Heijl A. (2007). Fluctuation of intraocular pressure and glaucoma progression in the early manifest glaucoma trial. Ophthalmology.

[20] Garway-Heath D.F., Crabb D.P., Bunce C., Lascaratos G., Amalfitano F., Anand N., Azuara-Blanco A., Bourne R.R., Broadway D.C., Cunliffe I.A. (2015). Latanoprost for open-angle glaucoma (UKGTS): A randomised, multicentre, placebo-controlled trial. Lancet.

[21] Sehi M., Grewal D.S., Goodkin M.L., Greenfield D.S. (2010). Reversal of retinal ganglion cell dysfunction after surgical reduction of intraocular pressure. Ophthalmology.

[22] Lee E.J., Kim T.-W., Weinreb R.N., Kim H. (2013). Reversal of lamina cribrosa displacement after intraocular pressure reduction in open-angle glaucoma. Ophthalmology.

[23] Bucolo C., Platania C.B.M., Drago F., Bonfiglio V., Reibaldi M., Avitabile T., Uva M. (2018). Novel therapeutics in glaucoma management. Curr. Neuropharmacol..

[24] Lusthaus J., Goldberg I. (2019). Current management of glaucoma. Med. J. Aust..

[25] Kaplan T.M., Sit A.J. (2022). Emerging drugs for the treatment of glaucoma: A review of phase II & III trials. Expert Opin. Emerg. Drugs.

[26] Civan M., Macknight A.D. (2004). The ins and outs of aqueous humor secretion. Exp. Eye Res..

[27] Acott T.S., Vranka J.A., Keller K.E., Raghunathan V., Kelley M.J. (2020). Normal and glaucomatous outflow regulation. Prog. Retin. Eye Res..

[28] Carreon T., van der Merwe E., Fellman R.L., Johnstone M., Bhattacharya S.K. (2017). Aqueous outflow—A continuum from trabecular meshwork to episcleral veins. Prog. Retin. Eye Res..

[29] Abu-Hassan D.W., Acott T.S., Kelley M.J. (2014). The trabecular meshwork: A basic review of form and function. J. Ocul. Biol..

[30] Buffault J., Labbé A., Hamard P., Brignole-Baudouin F., Baudouin C. (2020). The trabecular meshwork: Structure, function and clinical implications. A review of the literature. J. Fr. Ophtalmol..

[31] Van Zyl T., Yan W., McAdams A., Peng Y.R., Shekhar K., Regev A., Juric D., Sanes J.R. (2020). Cell atlas of aqueous humor outflow pathways in eyes of humans and four model species provides insight into glaucoma pathogenesis. Proc. Natl. Acad. Sci. USA.

[32] Patel G., Fury W., Yang H., Adler C., Wei Y., Ni M., Schmitt H., Hu Y., Yancopoulos G., Stamer W.D. (2020). Molecular taxonomy of human ocular outflow tissues defined by single-cell transcriptomics. Proc. Natl. Acad. Sci. USA.

[33] Overby D.R., Zhou E.H., Vargas-Pinto R., Pedrigi R.M., Fuchshofer R., Braakman S.T., Gupta R., Perkumas K.M., Sherwood J.M., Vahabikashi A. (2014). Altered mechanobiology of Schlemm’s canal endothelial cells in glaucoma. Proc. Natl. Acad. Sci. USA.

[34] Stamer W.D., Braakman S.T., Zhou E.H., Ethier C.R., Fredberg J.J., Overby D.R., Johnson M. (2015). Biomechanics of Schlemm’s canal endothelium and intraocular pressure reduction. Prog. Retin. Eye Res..

[35] Wang L.-Y., Su G.-Y., Wei Z.-Y., Zhang Z.J., Liang Q.F. (2020). Progress in the basic and clinical research on the Schlemm’s canal. Int. J. Ophthalmol..

[36] Lewczuk K., Jabłońska J., Konopińska J., Mariak Z., Rękas M. (2022). Schlemm’s canal: The outflow ‘vessel’. Acta Ophthalmol..

[37] Wiederholt M., Thieme H., Stumpff F. (2000). The regulation of trabecular meshwork and ciliary muscle contractility. Prog. Retin. Eye Res..

[38] Kim J.H., Caprioli J. (2018). Intraocular pressure fluctuation: Is it important?. J. Ophthalmic Vis. Res..

[39] Jasien J.V., Turner D.C., Girkin C.A., Downs J.C. (2018). Cyclic pattern of intraocular pressure (IOP) and transient IOP fluctuations in nonhuman primates measured with continuous wireless telemetry. Curr. Eye Res..

[40] Alvarado J., Murphy C., Juster R. (1984). Trabecular meshwork cellularity in primary open-angle glaucoma and nonglaucomatous normals. Ophthalmology.

[41] Grierson I., Howes R.C. (1987). Age-related depletion of the cell population in the human trabecular meshwork. Eye.

[42] Keller K.E., Peters D.M. (2022). Pathogenesis of glaucoma: Extracellular matrix dysfunction in the trabecular meshwork—A review. Clin. Exp. Ophthalmol..

[43] Kasetti R.B., Phan T.N., Millar J.C., Zode G.S. (2016). Expression of mutant myocilin induces abnormal intracellular accumulation of selected extracellular matrix proteins in the trabecular meshwork. Investig. Ophthalmol. Vis. Sci..

[44] Kasetti R.B., Maddineni P., Millar J.C., Clark A.F., Zode G.S. (2017). Increased synthesis and deposition of extracellular matrix proteins leads to endoplasmic reticulum stress in the trabecular meshwork. Sci. Rep..

[45] Yemanyi F., Vranka J., Raghunathan V.K. (2020). Crosslinked extracellular matrix stiffens human trabecular meshwork cells via dysregulating β-catenin and YAP/TAZ signaling pathways. Investig. Ophthalmol. Vis. Sci..

[46] Wang K., Johnstone M.A., Xin C., Padilla S., Vranka J.A., Acott T.S., Zhou K., Schwaner S.A., Wang R.K., Sulchek T. (2017). Estimating human trabecular meshwork stiffness by numerical modeling and advanced OCT imaging. Investig. Ophthalmol. Vis. Sci..

[47] Borrás T. (2017). A single gene connects stiffness in glaucoma and the vascular system. Exp. Eye Res..

[48] Morgan J.T., Raghunathan V.K., Chang Y.R., Murphy C.J., Russell P. (2015). The intrinsic stiffness of human trabecular meshwork cells increases with senescence. Oncotarget.

[49] Bermudez J.Y., Montecchi-Palmer M., Mao W., Clark A.F. (2017). Cross-linked actin networks (CLANs) in glaucoma. Exp. Eye Res..

[50] Patel P.D., Chen Y.L., Kasetti R.B., Maddineni P., Mayhew W., Millar J.C., Ellis D.Z., Sonkusare S.K., Zode G.S. (2021). Impaired TRPV4-eNOS signaling in trabecular meshwork elevates intraocular pressure in glaucoma. Proc. Natl. Acad. Sci. USA.

[51] Lakk M., Križaj D. (2021). TRPV4-Rho signaling drives cytoskeletal and focal adhesion remodeling in trabecular meshwork cells. Am. J. Physiol. Cell Physiol..

[52] Ryskamp D.A., Frye A.M., Phuong T.T., Yarishkin O., Jo A.O., Xu Y., Lakk M., Iuso A., Redmon S.N., Ambati B. (2016). TRPV4 regulates calcium homeostasis, cytoskeletal remodeling, conventional outflow and intraocular pressure in the mammalian eye. Sci. Rep..

[53] Yarishkin O., Phuong T.T.T., Baumann J.M., De Ieso M.L., Vazquez-Chona F., Rudzitis C.N., Sundberg C., Lakk M., Stamer W.D., Križaj D. (2021). Piezo1 channels mediate trabecular meshwork mechanotransduction and promote aqueous fluid outflow. J. Physiol..

[54] Dismuke W.M., Sharif N.A., Ellis D.Z. (2009). Human trabecular meshwork cell volume decrease by NO-independent soluble guanylate cyclase activators YC-1 and BAY-58-2667 involves the BKCa ion channel. Investig. Ophthalmol. Vis. Sci..

[55] Ellis D.Z., Sharif N.A., Dismuke W.M. (2010). Endogenous regulation of human Schlemm’s canal cell volume by nitric oxide signaling. Investig. Ophthalmol. Vis. Sci..

[56] Nathanson J.A. (1993). Nitric oxide and nitrovasodilators in the eye: Implications for ocular physiology and glaucoma. J. Glaucoma.

[57] Saccà S.C., Pascotto A., Camicione P., Capris P., Izzotti A. (2005). Oxidative DNA damage in the human trabecular meshwork: Clinical correlation in patients with primary open-angle glaucoma. Arch. Ophthalmol..

[58] He Y., Ge J., Tombran-Tink J. (2008). Mitochondrial defects and dysfunction in calcium regulation in glaucomatous trabecular meshwork cells. Investig. Ophthalmol. Vis. Sci..

[59] Ying Y., Xue R., Yang Y., Zhang S.X., Xiao H., Zhu H., Li J., Chen G., Ye Y., Yu M. (2021). Activation of ATF4 triggers trabecular meshwork cell dysfunction and apoptosis in POAG. Aging.

[60] Izzotti A., Longobardi M., Cartiglia C., Saccà S.C. (2011). Mitochondrial damage in the trabecular meshwork occurs only in primary open-angle glaucoma and in pseudoexfoliative glaucoma. PLoS ONE.

[61] De Groef L., Andries L., Siwakoti A., Geeraerts E., Bollaerts I., Noterdaeme L., Etienne I., Papageorgiou A.P., Stalmans I., Billen J. (2016). Aberrant collagen composition of the trabecular meshwork results in reduced aqueous humor drainage and elevated IOP in MMP-9 Null Mice. Investig. Ophthalmol. Vis. Sci..

[62] Adornetto A., Russo R., Parisi V. (2019). Neuroinflammation as a target for glaucoma therapy. Neural Regen. Res..

[63] Vernazza S., Tirendi S., Bassi A.M., Raverso C.E., Saccà S.C. (2020). Neuroinflammation in primary open-angle glaucoma. J. Clin. Med..

[64] Soto I., Howell G.R. (2014). The complex role of neuroinflammation in glaucoma. Cold Spring Harb. Perspect. Med..

[65] Wilson G.N., Inman D.M., Dengler-Crish C.M., Smith M.A., Crish S.D. (2015). Early pro-inflammatory cytokine elevations in the DBA/2J mouse model of glaucoma. J. Neuroinflamm..

[66] Yang X., Luo C., Cai J. (2011). Neurodegenerative and inflammatory pathway components linked to TNF-alpha/TNFR1 signaling in the glaucomatous human retina. Investig. Ophthalmol. Vis. Sci..

[67] Yerramothu P., Vijay A.K., Willcox M.D.P. (2018). Inflammasomes, the eye and anti-inflammasome therapy. Eye.

[68] Chintala S.K. (2006). The emerging role of proteases in retinal ganglion cell death. Exp. Eye Res..

[69] Chi W., Li F., Chen H., Wang Y., Zhu Y., Yang X., Zhu J., Wu F., Ouyang H., Ge J. (2014). Caspase-8 promotes NLRP1/NLRP3 inflammasome activation and IL-1β production in acute glaucoma. Proc. Natl. Acad. Sci. USA.

[70] Tribble J.R., Harder J.M., Williams P.A., John S.W.M. (2020). Ocular hypertension suppresses homeostatic gene expression in optic nerve head microglia of DBA/2 J mice. Mol. Brain.

[71] Burgoyne C.F., Downs J.C., Bellezza A.J., Suh J.K., Hart R.T. (2005). The optic nerve head as a biomechanical structure; a new paradigm for understanding the role of IOP-related stress and strain in the pathophysiology of glaucomatous optic nerve head damage. Prog. Retinal Eye Res..

[72] Downs J.C., Roberts M.D., Sigal I.A. (2011). Glaucomatous cupping of the lamina cribrosa: A review of the evidence for active progressive remodeling as a mechanism. Exp. Eye Res..

[73] Daguman I.J., Delfin M.S. (2018). Correlation of lamina cribosa and standard automated perimeter findings in glaucoma and non-glaucoma patients. J. Ophthal. Studies.

[74] Coudrillier B., Campbell I.C., Read A.T., Geraldes D.M., Vo N.T., Feola A., Mulvihill J., Albon J., Abel R.L., Ethier C.R. (2016). Effects of peripapillary scleral stiffening on the deformation of the lamina cribrosa. Investig. Ophthalmol. Vis. Sci..

[75] Jóhannesson G., Eklund A., Lindén C. (2018). Intracranial and intraocular pressure at the lamina cribrosa: Gradient effects. Curr. Neurol. NeuroSci. Rep..

[76] Wostyn P., De Groot V., Van Dam D., Audenaert K., Killer H.E., De Deyn P.P. (2015). Glaucoma and the role of cerebrospinal fluid dynamics. Investig. Ophthalmol. Vis. Sci..

[77] Park H.-Y.L., Lee K.-I.I., Lee K., Shin H.Y., Park C.K. (2015). Torsion of the optic nerve head is a prominent feature of normal-tension glaucoma. Investig. Ophthalmol. Vis. Sci..

[78] Nickells R.W., Howell G.R., Soto I., John S.W. (2012). Under pressure: Cellular and molecular responses during glaucoma, a common neurodegeneration with axonopathy. Annu. Rev. Neurosci..

[79] Evangelho K., Mogilevskaya M., Losada-Barragan M., Vargas-Sanchez J.K. (2019). Pathophysiology of primary open-angle glaucoma from a neuroinflammatory and neurotoxicity perspective: A review of the literature. Int. Ophthalmol..

[80] Fahy E.T., Chrysostomou V., Crowston J.G. (2016). Impaired axonal transport in glaucoma. Curr. Eye Res..

[81] Nuschke A.C., Farrell S.R., Levesque J.M., Chauhan B.C. (2015). Assessment of retinal ganglion cell damage in glaucomatous optic neuropathy: Axon transport, injury and soma loss. Exp. Eye Res..

[82] Li Y., Li D., Ying X., Khaw P.T., Raisman G. (2015). An energy theory of glaucoma. Glia.

[83] Osborne N.N., Nunez-Alvarez C., Joglar B., Del Olmo-Aguado S. (2016). Glaucoma: Focus on mitochondria in relation to pathogenesis and neuroprotection. Eur. J. Pharmacol..

[84] Eells J.T. (2019). Mitochondrial dysfunction in the aging retina. Biology.

[85] Silverman S.M., Kim B.J., Howell G.R., Miller J., John S.W., Wordinger R.J., Clark A.F. (2016). C1q propagates microglial activation and neurodegeneration in the visual axis following retinal ischemia/reperfusion injury. Mol. Neurodegener..

[86] Stasi K., Nagel D., Yang X., Wang R.F., Ren L., Podos S.M., Mittag T., Danias J. (2006). Complement component 1Q (C1Q) upregulation in retina of murine, primate, and human glaucomatous eyes. Investig. Ophthalmol. Vis. Sci..

[87] Nguyen D., Alavi M.V., Kim K.Y., Kang T., Scott R.T., Noh Y.H., Lindsey J.D., Wissinger B., Ellisman M.H., Weinreb R.N. (2011). A new vicious cycle involving glutamate excitotoxicity, oxidative stress and mitochondrial dynamics. Cell Death Dis..

[88] McElnea E.M., Quill B., Docherty N.G., Irnaten M., Siah W.F., Clark A.F., O’Brien C.J., Wallace D.M. (2011). Oxidative stress, mitochondrial dysfunction and calcium overload in human lamina cribrosa cells from glaucoma donors. Mol. Vis..

[89] Maddineni P., Kasetti R.B., Patel P.D., Millar J.C., Kiehlbauch C., Clark A.F., Zode G.S. (2020). CNS axonal degeneration and transport deficits at the optic nerve head precede structural and functional loss of retinal ganglion cells in a mouse model of glaucoma. Mol. Neurodegener..

[90] Skonieczna K., Grabska-Liberek I., Terelak-Borys B., Jamrozy-Witkowska A. (2014). Selected autoantibodies and normal-tension glaucoma. Med. Sci. Monit..

[91] Sanes J.R., Masland R.H. (2015). The types of retinal ganglion cells: Current status and implications for neuronal classification. Annu. Rev. Neurosci..

[92] Guo L., Moss S.E., Alexander R.A., Ali R.R., Fitzke F.W., Cordeiro M.F. (2005). Retinal ganglion cell apoptosis in glaucoma is related to intraocular pressure and IOP-induced effects on extracellular matrix. Investig. Ophthalmol. Vis. Sci..

[93] Resta V., Novelli E., Vozzi G., Scarpa C., Caleo M., Ahluwalia A., Solini A., Santini E., Parisi V., Di Virgilio F. (2007). Acute retinal ganglion cell injury caused by intraocular pressure spikes is mediated by endogenous extracellular ATP. Eur. J. Neurosci..

[94] Ou Y., Jo R.E., Ullian E.M., Wong R.O., Della Santina L. (2016). Selective vulnerability of specific retinal ganglion cell types and synapses after transient ocular hypertension. J. Neurosci..

[95] Della Santina L., Ou Y. (2017). Who’s lost first? Susceptibility of retinal ganglion cell types in experimental glaucoma. Exp. Eye Res..

[96] Chaphalkar R.M., Stankowska D.L., He S., Kodati B., Phillips N., Prah J., Yang S., Krishnamoorthy R.R. (2020). Endothelin-1 mediated decrease in mitochondrial gene expression and bioenergetics contribute to neurodegeneration of retinal ganglion cells. Sci. Rep..

[97] Bhandari A., Smith J.C., Zhang Y., Jensen A.A., Reid L., Goeser T., Fan S., Ghate D., Van Hook M.J. (2019). Early-stage ocular hypertension alters retinal ganglion cell synaptic transmission in the visual thalamus. Front. Cell. Neurosci..

[98] Dvoriantchikova G., Ivanov D. (2014). Tumor necrosis factor-alpha mediates activation of NF-κB and JNK signaling cascades in retinal ganglion cells and astrocytes in opposite ways. Eur. J. Neurosci..

[99] Tezel G., Yang X., Luo C., Kain A.D., Powell D.W., Kuehn M.H., Kaplan H.J. (2010). Oxidative stress and the regulation of complement activation in human glaucoma. Investig. Ophthalmol. Vis. Sci..

[100] Cooper M.L., Crish S.D., Inman D.M., Horner P.J., Calkins D.J. (2016). Early astrocyte redistribution in the optic nerve precedes axonopathy in the DBA/2J mouse model of glaucoma. Exp. Eye Res..

[101] Hollander H., Makarov F., Stefani F.H., Stone J. (1995). Evidence of constriction of optic axons at the lamina cribrosa in the normotensive eye in humans and other mammals. Ophthalmic Res..

[102] Kong Y.X., Crowston J.G., Vingrys A.J., Trounce I.A., Bui V.B. (2010). Functional changes in the retina during and after acute intraocular pressure elevation in mice. Investig. Ophthalmol. Vis. Sci..

[103] Ebneter A., Casson R.J., Wood J.P., Chidlow G. (2010). Microglial activation in the visual pathway in experimental glaucoma: Spatiotemporal characterization and correlation with axonal injury. Investig. Ophthalmol. Vis. Sci..

[104] Harwerth R.S., Quigley H.A. (2006). Visual field defects and retinal ganglion cell losses in patients with glaucoma. Arch Ophthalmol..

[105] Tu S., Li K., Ding X., Hu D., Li K., Ge J. (2019). Relationship between intraocular pressure and retinal nerve fibre thickness loss in a monkey model of chronic ocular hypertension. Eye.

[106] Xu G., Weinreb R.N., Leung C.K. (2014). Optic nerve head deformation in glaucoma: The temporal relationship between optic nerve head surface depression and retinal nerve fiber layer thinning. Ophthalmology.

[107] Sharif N.A. (2018). Glaucomatous optic neuropathy treatment options: The promise of novel therapeutics, techniques and tools to help preserve vision. Neural Regen. Res..

[108] Medeiros F.A., Weinreb R.N., Zangwill L.M., Alencar L.M., Sample P.A., Vasile C., Bowd C. (2008). Long-term intraocular pressure fluctuations and risk of conversion from ocular hypertension to glaucoma. Ophthalmology.

[109] Yucel Y.H., Zhang Q., Weinreb R.N., Kaufman P.L., Gupta N. (2001). Atrophy of relay neurons in magno- and parvocellular layers in the lateral geniculate nucleus in experimental glaucoma. Investig. Ophthalmol. Vis. Sci..

[110] Gupta N., Ly T., Zhang Q., Kaufman P.L., Weinreb R.N., Yücel Y.H. (2007). Chronic ocular hypertension induces dendrite pathology in the lateral geniculate nucleus of the brain. Exp. Eye Res..

[111] Trivedi V., Bang J.W., Parra C., Colbert M.K., O’Connell C., Arshad A., Faiq M.A., Conner I.P., Redfern M.S., Wollstein G. (2019). Widespread brain reorganization perturbs visuomotor coordination in early glaucoma. Sci. Rep..

[112] Van Hook M.J., Monaco C., Bierlein E.R., Smith J.C. (2021). Neuronal and synaptic plasticity in the visual thalamus in mouse models of glaucoma. Front. Cell. Neurosci..

[113] Hvozda Arana A.G., Lasagni Vitar R.M., Reides C.G., Calabró V., Marchini T., Lerner S.F., Evelson P.A., Ferreira S.M. (2021). Mitochondrial function is impaired in the primary visual cortex in an experimental glaucoma model. Arch. Biochem. Biophys..

[114] Yu L., Xie L., Dai C., Liang M., Zhao L., Yin X., Wang J. (2015). Progressive thinning of visual cortex in primary open-angle glaucoma of varying severity. PLoS ONE.

[115] Crabb D.P. (2016). A view on glaucoma—Are we seeing it clearly?. Eye.

[116] Sharif N.A., Odani-Kawabata N., Lu F., Pinchuk L. (2023). FP and EP2 prostanoid receptor agonist drugs and aqueous humor outflow devices for treating ocular hypertension and glaucoma. Exp. Eye Res..

[117] Klimko P., Sharif N.A. (2019). Discovery, characterization and clinical utility of prostaglandin agonists for treatment of glaucoma. Br. J. Pharmacol..

[118] Hollo G., Topouzis F., Fechtner R.D. (2014). Fixed-combination intraocular pressure-lowering therapy for glaucoma and ocular hypertension: Advantages in clinical practice. Expert Opin. Pharmacother..

[119] Asrani S., Bacharach J., Holland E., McKee H., Sheng H., Lewis R.A., Kopczynski C.C., Heah T. (2020). Fixed-dose combination of netarsudil and latanoprost in ocular hypertension and open-angle glaucoma: Pooled efficacy/safety analysis of phase 3 MERCURY-1 and -2. Adv. Ther..

[120] Nardi M., Casini G., Guidi G., Figus M. (2015). Emerging surgical therapy in the treatment of glaucoma. Prog. Brain Res..

[121] Ahmed I.I.K., Fea A., Au L., Ang R.E., Harasymowycz P., Jampel H.D., Samuelson T.W., Chang D.F., Rhee D.J., COMPARE Investigators (2020). A prospective randomized trial comparing Hydrus and iStent microinvasive glaucoma surgery implants for standalone treatment of open-angle glaucoma: The COMPARE Study. Ophthalmology.

[122] Lee R.M.H., Bouremel Y., Eames I., Brocchini S., Khaw P.T. (2020). Translating minimally invasive glaucoma surgery devices. Clin. Transl. Sci..

[123] Gazzard G., Konstantakopoulou E., Garway-Heath D., Adeleke M., Vickerstaff V., Ambler G., Hunter R., Bunce C., Nathwani N., Barton K. (2023). Laser in glaucoma and ocular hypertension (LiGHT) trial: Six-year results of primary selective laser trabeculoplasty versus eye drops for the treatment of glaucoma and ocular hypertension. Ophthalmology.

[124] Newman-Casey P.A., Robin A.L., Blachley T., Farris K., Heisler M., Resnicow K., Lee P.P. (2015). The most common barriers to glaucoma medication adherence. Ophthalmology.

[125] Alm A., Grierson I., Shields M.B. (2008). Side effects associated with prostaglandin analog therapy. Surv. Ophthalmol..

[126] Yeh P.H., Cheng Y.C., Shie S.S., Lee Y.S., Shen S.C., Chen H.S., Wu W.C., Su W.W. (2021). Brimonidine related acute follicular conjunctivitis: Onset time and clinical presentations, a long-term follow-up. Medicine.

[127] Sharif N.A. (2022). Neuropathology and therapeutics addressing glaucoma, a prevalent sight threatening retina-optic nerve-brain disease. OBM Neurobiol..

[128] Sharif N.A. (2018). *i*Drugs and *i*Devices discovery and development—Preclinical assays, techniques and animal model studies for ocular hypotensives and neuroprotectants. J. Ocular. Pharmacol. Ther..

[129] Rao P.V., Pattabiraman P.P., Kopczynski C. (2017). Role of the Rho GTPase/Rho kinase signaling pathway in pathogenesis and treatment of glaucoma: Bench to bedside research. Exp. Eye Res..

[130] Sharif N.A. (2022). Rho kinase inhibitor utility for glaucoma and optic neuropathy treatment: Enzymic activity, IOP-lowering and neuroprotection perspectives. Open Access J. Ophthalmol..

[131] Ramachandran C., Patil R.V., Sharif N.A., Srinivas S.P. (2011). Effect of elevated intracellular cAMP on actomyosin contraction in bovine trabecular meshwork cells. Investig. Ophthalmol. Vis. Sci..

[132] Ramachandran C., Patil R.V., Combrink K., Sharif N.A., Srinivas S.P. (2011). Rho-Rho kinase pathway in the actomyosin contraction and cell-matrix adhesion in immortalized human trabecular meshwork cells. Mol. Vis..

[133] Henderson A.J., Hadden M., Guo C., Douglas N., Decornez H., Hellberg M.R., Rusinko A., McLaughlin M., Sharif N., Drace C. (2010). 2,3-Diaminopyrines as rho kinase inhibitors. Bioorganic Med. Chem. Lett..

[134] Chen H.-H., Namil A., Severns B., Ward J., Kelly C.R., Drace C., McLaughlin M.A., Yacoub S., Li B., Patil R. (2014). In vivo optimization of 2,3-diaminopyrazine Rho kinase inhibitors. Bioorganic Med. Chem. Lett..

[135] Goldhagen B., Proia A.D., Epstein D.L., Rao P.V. (2012). Elevated levels of RhoA in the optic nerve head of human eyes with glaucoma. J. Glaucoma.

[136] Futakuchi A., Morimoto T., Ikeda Y., Tanihara H., Inoue T. (2020). Intraocular pressure-lowering effects of ripasudil in uveitic glaucoma, exfoliation glaucoma, and steroid-induced glaucoma patients: ROCK-S, a multicentre historical cohort study. Sci. Rep..

[137] Tanihara H., Inoue T., Yamamoto T., Kuwayama Y., Abe H., Suganami H., Araie M., K-115 Clinical Study Group (2015). Additive intraocular pressure-lowering effects of the rho kinase inhibitor ripasudil (K-115) combined with timolol or latanoprost: A report of 2 randomized clinical trials. JAMA Ophthalmol..

[138] Lin C.W., Sherman B., Moore L.A., Laethem C.L., Lu D.W., Pattabiraman P.P., Rao P.V., deLong M.A., Kopczynski C.C. (2018). Discovery and preclinical development of netarsudil, a novel ocular hypotensive agent for the treatment of glaucoma. J. Ocul. Pharmacol. Ther..

[139] Serle J.B., Katz L.J., McLaurin E., Heah T., Ramirez-Davis N., Usner D.W., Novack G.D., Kopczynski C.C., ROCKET-1 and ROCKET-2 Study Groups (2018). Two phase 3 clinical trials comparing the safety and efficacy of netarsudil to timolol in patients with elevated intraocular pressure: Rho kinase elevated IOP treatment trial 1 and 2 (ROCKET-1 and ROCKET-2). Am. J. Ophthalmol..

[140] Singh I.P., Fechtner R.D., Myers J.S., Kim T., Usner D.W., McKee H., Sheng H., Lewis R.A., Heah T., Kopczynski C.C. (2020). Pooled efficacy and safety profile of netarsudil ophthalmic solution 0.02% in patients with open-angle glaucoma or ocular hypertension. J. Glaucoma.

[141] Rashad R., Zhu C., Kupcha A.C., Distefano A.G., Kefella H., Desai M.A. (2022). Partial stenosis and complete punctal closure following topical netarsudil use for glaucoma. J. Glaucoma.

[142] Sit A.J., Gupta D., Kazemi A., McKee H., Challa P., Liu K.C., Lopez J., Kopczynski C., Heah T. (2021). Netarsudil Improves Trabecular Outflow Facility in Patients with Primary Open Angle Glaucoma or Ocular Hypertension: A Phase 2 Study. Am. J. Ophthalmol..

[143] Walters T.R., Ahmed I.I.K., Lewis R.A., Usner D.W., Lopez J., Kopczynski C.C., Heah T., MERCURY-2 Study Group (2019). Once-Daily Netarsudil/Latanoprost Fixed-Dose Combination for Elevated Intraocular Pressure in the Randomized Phase 3 MERCURY-2 Study. Ophthalmol Glaucoma..

[144] Ren R., Li G., Le T.D., Kopczynski C., Stamer W.D., Gong H. (2016). Netarsudil increases outflow facility in human eyes through multiple mechanisms. Investig. Ophthalmol. Vis. Sci..

[145] Van de Velde S., De Groef L., Stalmans I., Moons L., Van Hove I. (2015). Towards axonal regeneration and neuroprotection in glaucoma: Rho kinase inhibitors as promising therapeutics. Prog. Neurobiol..

[146] Sagawa H., Terasak H., Nakamura M., Ichikawa M., Ata T., Tokita Y., Watanabe M. (2007). A novel rock inhibitor, Y-39983, promotes regeneration of crushed axons of retinal ganglion cells into the optic nerve of adult cats. Exp. Neurol..

[147] Shaw P.X., Sang A., Wang Y., Ho D., Douglas C., Dia L., Goldberg J.L. (2017). Topical administration of a ROCK/NET inhibitor promotes retinal ganglion cell survival and axon regeneration after optic nerve injury. Exp. Eye Res..

[148] Li G., Lee C., Read A.T., Wang K., Ha J., Kuhn M., Navarro I., Cui J., Young K., Gorijavolu R. (2021). Anti-fibrotic activity of a rho-kinase inhibitor restores outflow function and intraocular pressure homeostasis. Elife.

[149] Pattabiraman P.P., Rinkoski T., Poeschla E., Proia A. (2015). RhoA GTPase-induced ocular hypertension in a rodent model is associated with increased fibrogenic activity in the trabecular meshwork. Am. J. Pathol..

[150] Yamashita K., Kotani Y., Nakajima Y., Shimazawa M., Yoshimura S., Nakashima S., Iwama T., Hara H. (2007). Fasudil, a rho kinase (ROCK) inhibitor, protects against ischemic neuronal damage in vitro and in vivo by acting directly on neurons. Brain Res..

[151] Tokushige H., Waki M., Takayama Y., Tanihara H. (2011). Effects of Y-39983, a selective rho-associated protein kinase inhibitor, on blood flow in optic nerve head in rabbits and axonal regeneration of retinal ganglion cells in rats. Curr. Eye Res..

[152] Bastia E., Toris C.B., Brambilla S., Galli C., Almirante N., Bergamini M.V.W., Masini E., Sgambellone S., Unser A.M., Ahmed F. (2021). NCX 667, a novel nitric oxide donor, lowers intraocular pressure in rabbits, dogs, and non-human primates and enhances TGFβ2-induced outflow in HTM/HSC constructs. Investig. Ophthalmol. Vis. Sci..

[153] Nathanson J.A., McKee M. (1995). Identification of an extensive system of nitric oxide-producing cells in the ciliary muscle and outflow pathway of the human eye. Investig. Ophthalmol. Vis. Sci..

[154] Nathanson J.A., McKee M. (1995). Alterations of ocular nitric oxide synthase in human glaucoma. Investig. Ophthalmol. Vis. Sci..

[155] Cavet M.E., DeCory H.H. (2018). The role of nitric oxide in the intraocular pressure lowering efficacy of latanoprostene bunod: Review of nonclinical studies. J. Ocul. Pharmacol. Ther..

[156] Kondkar A.A., Azad T.A., Sultan T., Osman E.A., Almobarak F.A., Al-Obeidan S.A. (2020). Association of endothelial nitric oxide synthase (NOS3) gene polymorphisms with primary open-angle glaucoma in a Saudi cohort. PLoS ONE.

[157] Reina-Torres E., De Ieso M.L., Pasquale L.R., Madekurozwa M., van Batenburg-Sherwood J., Overby D.R., Stamer W.D. (2021). The vital role for nitric oxide in intraocular pressure homeostasis. Prog. Retin. Eye Res..

[158] Ellis D., Scheibler L., Sharif N.A. (2017). Prostaglandin Conjugates and Derivatives for Treating Glaucoma and Ocular Hypertension. U.S. Patent.

[159] Shahidullah M., Mandal A., Wei G., Delamere N.A. (2014). Nitric oxide regulation of Na, K-ATPase activity in ocular ciliary epithelium involves Src family kinase. J. Cell Physiol..

[160] Radell J.E., Sharma H.K., Auyeung K.L., Paul M.E., Gagliuso D.J., Chadha N., Tsai J.C., Serle J.B. (2021). Two-Year Experience With Latanoprostene Bunod in Clinical Practice. J. Glaucoma.

[161] Xu D., Wu F., Yu Y., Lou X., Ye M., Zhang H., Zhao Y. (2022). Sympathetic activation leads to Schlemm’s canal expansion via increasing vasoactive intestinal polypeptide secretion from trabecular meshwork. Exp. Eye Res..

[162] Roddy G.W., Roy Chowdhury U., Anderson K.J., Rinkoski T.A., Hann C.R., Chiodo V.A., Smith W.C., Fautsch M.P. (2022). Transgene expression of Stanniocalcin-1 provides sustained intraocular pressure reduction by increasing outflow facility. PLoS ONE.

[163] Weinreb R.N., Scassellati Sforzolini B., Vittitow J., Liebmann J. (2016). Latanoprostene Bunod 0.024% versus Timolol Maleate 0.5% in Subjects with Open-Angle Glaucoma or Ocular Hypertension: The APOLLO Study. Ophthalmology.

[164] Weinreb R.N., Liebmann J.M., Martin K.R., Kaufman P.L., Vittitow J.L. (2018). Latanoprostene Bunod 0.024% in Subjects With Open-angle Glaucoma or Ocular Hypertension: Pooled Phase 3 Study Findings. J. Glaucoma.

[165] Fingeret M., Gaddie I.B., Bloomenstein M. (2019). Latanoprostene bunod ophthalmic solution 0.024%: A new treatment option for open-angle glaucoma and ocular hypertension. Clin. Exp. Optom..

[166] Hoy S.M. (2018). Latanoprostene Bunod Ophthalmic Solution 0.024%: A Review in Open-Angle Glaucoma and Ocular Hypertension. Drugs.

[167] Addis V.M., Miller-Ellis E. (2018). Latanoprostene bunod ophthalmic solution 0.024% in the treatment of open-angle glaucoma: Design, development, and place in therapy. Clin. Ophthalmol..

[168] Sharif N.A., Williams G.W., Kelly C.R. (2001). Bimatoprost and its free acid are prostaglandin FP receptor agonists. Eur. J. Pharmacol..

[169] Sharif N.A., Kelly C.R., Crider J.Y. (2003). Human trabecular meshwork cell responses induced by bimatoprost, travoprost, unoprostone, and other FP prostaglandin receptor agonist analogues. Investig. Ophthalmol. Vis. Sci..

[170] Kelly C.R., Williams G.W., Sharif N.A. (2003). Real-time intracellular Ca^2+^ mobilization by travoprost acid, bimatoprost, unoprostone, and other analogs via endogenous mouse, rat, and cloned human FP prostaglandin receptors. J. Pharmacol. Exp. Ther..

[171] Camras C.B., Sharif N.A., Wax M.B., Stjernschantz J. (2008). Bimatoprost, the prodrug of a prostaglandin analogue. Br. J. Ophthalmol..

[172] Lee S.S., Dibas M., Almazan A., Robinson M.R. (2019). Dose-response of intracameral bimatoprost sustained-release implant and topical bimatoprost in lowering intraocular pressure. J. Ocul. Pharmacol. Ther..

[173] Lee S.S., Burke J., Shen J., Almazan A., Orilla W., Hughes P., Zhang J., Li H., Struble C., Miller P.E. (2018). Bimatoprost sustained-release intracameral implant reduces episcleral venous pressure in dogs. Vet. Ophthalmol..

[174] Brandt J.D., Sall K., DuBiner H., Benza R., Alster Y., Walker G., Semba C.P., Collaborators (2016). Six-month intraocular pressure reduction with a topical bimatoprost ocular insert: Results of a phase II randomized controlled study. Ophthalmology.

[175] Bacharach J., Tatham A., Ferguson G., Belalcázar S., Thieme H., Goodkin M.L., Chen M.Y., Guo Q., Liu J., Robinson M.R. (2021). Phase 3, randomized, 20-month study of the efficacy and safety of bimatoprost implant in patients with open-angle glaucoma and ocular hypertension (ARTEMIS 2). Drugs.

[176] Medeiros F.A., Sheybani A., Shah M.M., Rivas M., Bai Z., Werts E., Ahmed I.I.K., Craven E.R. (2022). Single administration of intracameral bimatoprost implant 10 µg in patients with open-angle glaucoma or ocular hypertension. Ophthalmol. Ther..

[177] Shirley M. (2020). Bimatoprost implant: First approval. Drugs Aging.

[178] Hellberg M.R., McLaughlin M.A., Sharif N.A., DeSantis L., Dean T.R., Kyba E.P., Bishop J.E., Klimko P.G., Zinke P.W., Selliah R.D. (2002). Identification and characterization of the ocular hypotensive efficacy of travoprost, a potent and selective FP prostaglandin receptor agonist, and AL-6598, a DP prostaglandin receptor agonist. Surv. Ophthalmol..

[179] Sharif N.A., Williams G.W., Crider J.Y., Xu S.X., Davis T.L. (2004). Molecular pharmacology of the DP/EP2 class prostaglandin AL-6598 and quantitative autoradiographic visualization of DP and EP2 receptor sites in human eyes. J. Ocul. Pharmacol. Ther..

[180] Nilsson S.F., Drecoll E., Lutjen-Drecoll E., Toris C.B., Krauss A.H., Kharlamb A., Nieves A., Guerra T., Woodward D.F. (2006). The prostanoid EP2 receptor agonist butaprost increases uveoscleral outflow in the cynomolgus monkey. Investig. Ophthalmol. Vis. Sci..

[181] Prasanna G., Carreiro S., Anderson S., Gukasyan H., Sartnurak S., Younis H., Gale D., Xiang C., Wells P., Dinh D. (2011). Effect of PF-04217329 a prodrug of a selective prostaglandin EP(2) agonist on intraocular pressure in preclinical models of glaucoma. Exp. Eye Res..

[182] Schachar R.A., Raber S., Courtney R., Zhang M. (2011). A phase 2, randomized, dose-response trial of taprenepag isopropyl (PF-04217329) versus latanoprost 0.005% in open-angle glaucoma and ocular hypertension. Curr. Eye Res..

[183] Aguirre S.A., Huang W., Prasanna G., Jessen B. (2009). Corneal neovascularization and ocular irritancy responses in dogs following topical ocular administration of an EP4-prostaglandin E2 agonist. Toxicol. Pathol..

[184] Krauss A.H., Woodward D.F., Chen J., Gibson L.L., Lai R.K., Protzman C.E., Shan T., Williams L.S., Gac T.S., Burk R.M. (1995). AGN 191976: A novel thromboxane A2-mimetic with ocular hypotensive properties. J. Ocul. Pharmacol. Ther..

[185] Yamane S., Karakawa T., Nakayama S., Nagai K., Moriyuki K., Neki S., Suto F., Kambe T., Hirota Y., Kawabata K. (2015). IOP-lowering effect of ONO-9054, a novel dual agonist of prostanoid EP3 and FP receptors, in monkeys. Investig. Ophthalmol. Vis. Sci..

[186] Iwamura R., Tanaka M., Okanari E., Kirihara T., Odani-Kawabata N., Shams N., Yoneda K. (2018). Identification of a Selective, Non-Prostanoid EP2 Receptor Agonist for the Treatment of Glaucoma: Omidenepag and its Prodrug Omidenepag Isopropyl. J. Med. Chem..

[187] Kirihara T., Taniguchi T., Yamamura K., Iwamura R., Yoneda K., Odani-Kawabata N., Shimazaki A., Matsugi T., Shams N., Zhang J.Z. (2018). Pharmacologic Characterization of Omidenepag Isopropyl, a Novel Selective EP2 Receptor Agonist, as an Ocular Hypotensive Agent. Investig. Ophthalmol. Vis. Sci..

[188] Fuwa M., Toris C.B., Fan S., Taniguchi T., Ichikawa M., Odani-Kawabata N., Iwamura R., Yoneda K., Matsugi T., Shams N.K. (2018). Effects of a novel selective EP2 receptor agonist, omidenepag isopropyl, on aqueous humor dynamics in laser-induced ocular hypertensive monkeys. J. Ocul. Pharmacol. Ther..

[189] Crider J.Y., Griffin B.W., Sharif N.A. (1998). Prostaglandin-stimulated adenylyl cyclase activity via a pharmacologically-defined EP2 receptor in human NPE cells. J. Ocular. Pharmacol. Ther..

[190] Crider J.Y., Sharif N.A. (2001). Functional pharmacological evidence for EP2 and EP4 prostanoid receptors in immortalized human trabecular meshwork and non-pigmented ciliary epithelial cells. J. Ocul. Pharmacol. Ther..

[191] Schlotzer-Schrehardt U., Zenkel M., Nusing R.M. (2002). Expression and localization of FP and EP prostanoid receptor subtypes in human ocular tissues. Investig. Ophthalmol. Vis. Sci..

[192] Rosch S.R., Ramer R., Brune K., Hinz B. (2005). Prostaglandin E2 induces cyclooxygenase-2 expression in human non-pigmented ciliary epithelial cells through activation of p38 and p42/44 mitogen-activated protein kinases. Biochem. Biophys Res. Commun..

[193] Yousufzai S.Y., Ye Z., Abdel-Latif A.A. (1996). Prostaglandin F2 alpha and its analogs induce release of endogenous prostaglandins in iris and ciliary muscles isolated from cat and other mammalian species. Exp. Eye Res..

[194] Bergh K., Wentzel P., Stjernschantz J. (2002). Production of prostaglandin E(2) by iridial melanocytes exposed to latanoprost acid, a prostaglandin F(2 alpha) analogue. J. Ocul. Pharmacol. Ther..

[195] Uchida T., Shimizu S., Yamagishi R., Tokuoka S.M., Kita Y., Honjo M., Aihara M. (2021). Mechanical stretch induces Ca(^2+^) influx and extracellular release of PGE2 through Piezo1 activation in trabecular meshwork cells. Sci. Rep..

[196] Chen J., Woodward D.F. (1992). Prostanoid-induced relaxation of precontracted cat ciliary muscle is mediated by EP2 and DP receptors. Investig. Ophthalmol. Vis. Sci..

[197] Goh Y., Hotehama Y., Mishima H.K. (1995). Characterization of ciliary muscle relaxation induced by various agents in cats. Investig. Ophthalmol. Vis. Sci..

[198] Krauss A.H., Wiederholt M., Sturm A., Woodward D.F. (1997). Prostaglandin effects on the contractility of bovine trabecular meshwork and ciliary muscle. Exp. Eye Res..

[199] Wiederholt M., Sturm A., Lepple-Wienhues A. (1994). Relaxation of trabecular meshwork and ciliary muscle by release of nitric oxide. Investig. Ophthalmol. Vis. Sci..

[200] Anthony T.L., Lindsey J.D., Aihara M., Weinreb R.N. (2001). Detection of prostaglandin EP(1), EP(2), and FP receptor subtypes in human sclera. Investig. Ophthalmol. Vis. Sci..

[201] Nakamura N., Honjo M., Yamagishi R., Igarashi N., Sakata R., Aihara M. (2021). Effects of selective EP2 receptor agonist, omidenepag, on trabecular meshwork cells, Schlemm’s canal endothelial cells and ciliary muscle contraction. Sci. Rep..

[202] Kumon M., Fuwa M., Shimazaki A., Odani-Kawabata N., Iwamura R., Yoneda K., Kato M. (2023). Downregulation of COL12A1 and COL13A1 by a selective EP2 receptor agonist, omidenepag, in human trabecular meshwork cells. PLoS ONE.

[203] Kalouche G., Boucher C., Coste A., Debussche L., Orsini C., Baudouin C., Debeir T., Vigé X., Rostène W. (2016). Prostaglandin EP2 receptor signaling protects human trabecular meshwork cells from apoptosis induced by ER stress through down-regulation of p53. Biochim. Et Biophys. Acta (BBA)-Mol. Cell Res..

[204] Kalouche G., Beguier F., Bakria M., Melik-Parsadaniantz S., Leriche C., Debeir T., Rostène W., Baudouin C., Vigé X. (2016). Activation of Prostaglandin FP and EP2 Receptors Differently Modulates Myofibroblast Transition in a Model of Adult Primary Human Trabecular Meshwork Cells. Investig. Ophthalmol. Vis. Sci..

[205] Aihara M., Lu F., Kawata H., Tanaka Y., Yamamura K., Odani-Kawabata N., Shams N.K. (2019). Pharmacokinetics, safety, and intraocular pressure-lowering profile of omidenepag isopropyl, a selective, non-prostaglandin, prostanoid EP2 receptor agonist, in healthy Japanese and Caucasian volunteers (Phase I Study). J. Ocul. Pharmacol. Ther..

[206] Aihara M., Lu F., Kawata H., Iwata A., Liu K., Odani-Kawabata N., Shams N.K. (2019). Phase 2, randomized, dose-finding studies of omidenepag isopropyl, a selective EP2 agonist, in patients with primary open-angle glaucoma or ocular hypertension. J. Glaucoma.

[207] Aihara M., Lu F., Kawata H., Iwata A., Odani-Kawabata N. (2021). Twelve-month efficacy and safety of omidenepag isopropyl, a selective EP2 agonist, in open-angle glaucoma and ocular hypertension: The RENGE study. Jpn. J. Ophthalmol..

[208] Aihara M., Lu F., Kawata H., Iwata A., Odani-Kawabata N., Shams N.K. (2020). Omidenepag isopropyl versus latanoprost in primary open-angle glaucoma and ocular hypertension: The phase 3 AYAME study. Am. J. Ophthalmol..

[209] Aihara M., Ropo A., Lu F., Kawata H., Iwata A., Odani-Kawabata N., Shams N.K. (2020). Intraocular pressure-lowering effect of omidenepag isopropyl in latanoprost non-/low-responder patients with primary open-angle glaucoma or ocular hypertension: The FUJI study. Jpn. J. Ophthalmol..

[210] McLaurin E.B., Tepedino M.E. Omidenepag is Opropyl 0.002% Significantly Lowers Iop in Latanoprost Low/Non-Responders with POAG or OHT: Phase 3 SPECTRUM 5 Study. Proceedings of the World Ophthalmology Congress.

[211] Matsuo M., Matsuoka Y., Tanito M. (2022). Efficacy and patient tolerability of omidenepag isopropyl in the treatment of glaucoma and ocular hypertension. Clin. Ophthalmol..

[212] Miki A., Miyamoto E., Ishida N., Shii D., Hori K., LESPOIR Research Group (2022). Efficacy and Safety of Omidenepag Isopropyl 0.002% Ophthalmic Solution: A Retrospective Analysis of Real-World Data in Japan. Adv. Ther..

[213] Nakazawa T., Takahashi K., Kuwayama Y., Nomura A., Shimada F. (2022). Interim Results of Post-Marketing Observational Study of Omidenepag Isopropyl for Glaucoma and Ocular Hypertension in Japan. Adv. Ther..

[214] Fuwa M., Shimazaki A., Odani-Kawabata N., Kirihara T., Taniguchi T., Iwamura R., Yoneda K., Kato M., Morishima K., Shams N.K. (2021). Additive intraocular pressure-lowering effects of a novel selective EP2 receptor agonist, omidenepag isopropyl, combined with existing antiglaucoma agents in conscious ocular normotensive monkeys. J. Ocul. Pharmacol. Ther..

[215] Wistrand P.J., Stjernschantz J., Olsson K. (1997). The incidence and time-course of latanoprost-induced iridial pigmentation as a function of eye color. Surv. Ophthalmol..

[216] Kucukevcilioglu M., Bayer A., Uysal Y., Altinsoy H.I. (2014). Prostaglandin associated periorbitopathy in patients using bimatoprost, latanoprost and travoprost. Clin. Exp. Ophthalmol..

[217] Terao E., Nakakura S., Nagata Y., Dote S., Tabuchi H., Kiuchi Y. (2020). Evaluation of patterns and correlations of the degree of conjunctival hyperemia induced by omidenepag isopropyl 0.002% and ripasudil 0.4%. Cureus.

[218] Esaki Y., Katsuta O., Kamio H., Noto T., Mano H., Iwamura R., Yoneda K., Odani-Kawabata N., Morishima K., Shams N.K. (2020). The antiglaucoma agent and EP2 receptor agonist omidenepag does not affect eyelash growth in mice. J. Ocul. Pharmacol. Ther..

[219] Inoue K., Shiokawa M., Katakura S., Tsuruoka M., Kunimatsu-Sanuki S., Shimizu K., Ishida K., Tomita G. (2022). Periocular adverse reactions to omidenepag isopropyl. Am. J. Ophthalmol..

[220] Liu P., Wang F., Song Y., Wang M., Zhang X. (2022). Current situation and progress of drugs for reducing intraocular pressure. Ther. Adv. Chronic. Dis..

[221] Wang T., Cao L., Jiang Q., Zhang T. (2021). Topical medication therapy for glaucoma and ocular hypertension. Front. Pharmacol..

[222] Donegan R.K., Lieberman R.L. (2016). Discovery of molecular therapeutics for glaucoma: Challenges, successes, and promising directions. J. Med. Chem..

[223] Bouhenni R.A., Dunmire J., Sewell A., Edward D.P. (2012). Animal models of glaucoma. J. Biomed. Biotechnol..

[224] Harada C., Kimura A., Guo X., Namekata K., Harada T. (2019). Recent advances in genetically modified animal models of glaucoma and their roles in drug repositioning. Br. J. Ophthalmol..

[225] Struebing F.L., Geisert E.E. (2015). What animal models can tell us about glaucoma. Prog. Mol. Biol. Transl. Sci..

[226] Sharif N.A. (2020). Discovery to launch of anti-allergy (Emadine; Patanol/Pataday/Pazeo) and anti-glaucoma (Travatan; Simbrinza) ocular drugs, and generation of novel pharmacological tools such as AL-8810. ACS Pharmacol. Transl. Sci..

[227] Woodward D.F., Wang J.W., Stamer W.D., Lütjen-Drecoll E., Krauss A.H., Toris C.B. (2019). Antiglaucoma EP2 agonists: A long road that led somewhere. J. Ocul. Pharmacol. Ther..

[228] Ibrahim M.M., Maria D.N., Mishra S.R., Guragain D., Wang X., Jablonski M.M. (2019). Once daily pregabalin eye drops for management of glaucoma. ACS Nano.

[229] Honjo M., Igarashi N., Kurano M., Yatomi Y., Igarashi K., Kano K., Aoki J., Weinreb R.N., Aihara M. (2018). Autotaxin–lysophosphatidic acid pathway in intraocular pressure regulation and glaucoma subtypes. Investig. Ophthalmol. Vis. Sci..

[230] Martínez T., González M.V., Roehl I., Wright N., Pañeda C., Jiménez A.I. (2014). In vitro and in vivo efficacy of SYL040012, a novel siRNA compound for treatment of glaucoma. Mol. Ther..

[231] Pfeiffer N., Voykov B., Renieri G., Bell K., Richter P., Weigel M., Thieme H., Wilhelm B., Lorenz K., Feindor M. (2017). First-in-human phase I study of ISTH0036, an antisense oligonucleotide selectively targeting transforming growth factor beta 2 (TGF-β2), in subjects with open-angle glaucoma undergoing glaucoma filtration surgery. PLoS ONE.

[232] Sun D., Zhan Z., Zeng R., Liu X., Wang B., Yang F., Huang S., Li Y., Yang Z., Su Y. (2022). Long-term and potent IOP-lowering effect of IκBα-siRNA in a nonhuman primate model of chronic ocular hypertension. iScience.

[233] O’Callaghan J., Crosbie D.E., Cassidy P.S., Sherwood J.M., Flügel-Koch C., Lütjen-Drecoll E., Humphries M.M., Reina-Torres E., Wallace D., Kiang A.S. (2017). Therapeutic potential of AAV-mediated MMP-3 secretion from corneal endothelium in treating glaucoma. Hum. Mol. Genet..

[234] Jain A., Zode G., Kasetti R.B., Ran F.A., Yan W., Sharma T.P., Bugge K., Searby C.C., Fingert J.H., Zhang F. (2017). CRISPR-Cas9-based treatment of myocilin associated glaucoma. Proc. Natl. Acad. Sci. USA.

[235] Wu J., Bell O.H., Copland D.A., Young A., Pooley J.R., Maswood R., Evans R.S., Khaw P.T., Ali R.R., Dick A.D. (2020). Gene therapy for glaucoma by ciliary body aquaporin 1 disruption using CRISPR-Cas. Mol. Ther..

[236] Levin L.A., Patrick C., Choudry N.B., Sharif N.A., Goldberg J.L. (2022). Neuroprotection in neurodegenerations of the brain and eye: Lessons from the past and directions for the future. Front. Neurol..

[237] Howell G.R., MacNicoll K.H., Braine C.E., Soto I., Macalinao D.G., Sousa G.L., John S.W. (2014). Combinatorial targeting of early pathways profoundly inhibits neurodegeneration in a mouse model of glaucoma. Neurobiol. Dis..

[238] He S., Stankowska D.L., Ellis D.Z., Krishnamoorthy R.R., Yorio T. (2018). Targets of neuroprotection in glaucoma. J. Ocul. Pharmacol. Ther..

[239] Boia R., Ruzafa N., Aires I.D., Pereiro X., Ambrósio A.F., Vecino E., Santiago A.R. (2020). Neuroprotective strategies for retinal ganglion cell degeneration: Current status and challenges ahead. Int. J. Mol. Sci..

[240] Williams P.A., Harder J.M., Cochran K.E., Philip V.M., Porciatti V., Smithies O., John S.W.M. (2017). Vitamin B3 modulates mitochondrial vulnerability and prevents glaucoma in aged mice. Science.

[241] Komáromy A.M., Koehl K.L., Park S.A. (2021). Looking into the future: Gene and cell therapies for glaucoma. Vet. Ophthalmol..

[242] Zhu W., Jain A., Gramlich O.W., Tucker B.A., Sheffield V.C., Kuehn M.H. (2017). Restoration of aqueous humor outflow following transplantation of iPSC derived trabecular meshwork cells in a transgenic mouse model of glaucoma. Investig. Ophthalmol. Vis. Sci..

[243] Coulon S.J., Schuman J.S., Du Y., Bahrani Fard M.R., Ethier C.R., Stamer W.D. (2022). A novel glaucoma approach: Stem cell regeneration of the trabecular meshwork. Prog. Retin. Eye Res..

[244] Zhang J., Wu S., Jin Z.B., Wang N. (2021). Stem cell-based regeneration and restoration for retinal ganglion cell: Recent advancements and current challenges. Biomolecules.

[245] Sharif N.A. Electrical, electromagnetic, ultrasound wave therapies and electronic implants for neuronal rejuvenation, neuroprotection, axonal regeneration and IOP reduction. J. Ocul. Pharmacol. Ther..

[246] Batabyal S., Kim S., Wright W., Mohanty S. (2021). Layer-specific nanophotonic delivery of therapeutic opsin-encoding genes into retina. Exp. Eye Res..

[247] Wood E.H., Kreymerman A., Kowal T., Buickians D., Sun Y., Muscat S., Mercola M., Moshfeghi D.M., Goldberg J.L. (2022). Cellular and subcellular optogenetic approaches towards neuroprotection and vision restoration. Prog. Retin. Eye Res..

[248] Verta R., Saccu G., Tanzi A., Grange C., Buono L., Fagoonee S., Deregibus M.C., Camussi G., Scalabrin S., Nuzzi R. (2023). Phenotypic and functional characterization of aqueous humor derived extracellular vesicles. Exp. Eye Res..

[249] Williams P.A., Harder J.M., Foxworth N.E., Cardozo B.H., Cochran K.E., John S.W.M. (2017). Nicotinamide and WLDS Act Together to Prevent Neurodegeneration in Glaucoma. Front Neurosci..

[250] Hui F., Tang J., Williams P.A., Hadoux X., Casson R.J., Coote M., Trounce I.A., Martin K.R., van Wijngaarden P., Crowston J.G. (2020). Improvement in inner retinal function in glaucoma with nicotinamide (vitamin B3) supplementation: A crossover randomized clinical trial. Clin. Exp. Ophthalmol..

[251] Gaboriau T., Dubois R., Foucque B., Malet F., Schweitzer C. (2023). 24-Hour monitoring of intraocular pressure fluctuations using a contact lens sensor: Diagnostic performance for glaucoma progression. Investig. Ophthalmol. Vis. Sci..

[252] Cordeiro M.F., Hill D., Patel R., Corazza P., Maddison J., Younis S. (2022). Detecting retinal cell stress and apoptosis with DARC: Progression from lab to clinic. Prog. Retin. Eye Res..

[253] Wong D., Chua J., Lin E., Tan B., Yao X., Chong R., Sng C., Lau A., Husain R., Aung T. (2020). Focal structure-function relationships in primary open-angle glaucoma using OCT and OCT-A measurements. Investig. Ophthalmol. Vis. Sci..

[254] Zheng C., Johnson T.V., Garg A., Boland M.V. (2019). Artificial intelligence in glaucoma. Curr. Opin. Ophthalmol..

[255] Kasi A., Faiq M.A., Chan K.C. (2019). In vivo imaging of structural, metabolic and functional brain changes in glaucoma. Neural Regen. Res..

[256] Torres L.A., Hatanaka M. (2019). Correlating structural and functional damage in glaucoma. J. Glaucoma.

